# Guiding treatment decisions in early breast cancer: A model-based comparison of the OncotypeDX and MammaPrint tests

**DOI:** 10.1016/j.breast.2026.104698

**Published:** 2026-01-12

**Authors:** Frank Doornkamp, Liesbeth C. de Wreede, Elfi Verheul, Agnes Jager, Ewout W. Steyerberg

**Affiliations:** a Department of Biomedical Data Sciences, Leiden University Medical Center, Leiden, the Netherlands; b Department of Surgery, Erasmus MC Cancer Institute, Rotterdam, the Netherlands; c Scientific Bureau, Dutch Institute for Clinical Auditing, Leiden, the Netherlands; d Department of Medical Oncology, Erasmus University Medical Centre, Rotterdam, the Netherlands; e Julius Center for Health Sciences and Primary Care, University Medical Center Utrecht, Utrecht, the Netherlands

**Keywords:** Clinical usefulness, Net benefit, MammaPrint, OncotypeDX, Decision analysis

## Abstract

**Introduction:**

Genomic tests may improve chemotherapy allocation in early-stage breast cancer beyond traditional clinical factors. We compared the clinical usefulness of two multi-genomic tests, MammaPrint and OncotypeDX, in guiding adjuvant chemotherapy decisions.

**Methods:**

The MINDACT and TAILORx trials provided prospective validation for the MammaPrint and OncotypeDX tests, respectively. We generated two synthetic cohorts to evaluate both tests in both trial contexts. Chemotherapy was assumed to be indicated if it was expected to reduce the risk of an event by at least 5 %, defining events as 10-year distant metastases or breast cancer–related death. We compared treatment decision making informed by clinical risk information alone versus clinical information plus either the MammaPrint (dichotomous score: high/low risk) or OncotypeDX test (dichotomous and continuous scores). These strategies were evaluated using Net Benefit: a weighted difference of preventing events through treatment and the number of treatments given.

**Results:**

Treatment decision-making informed by clinical information alone would result in a positive balance between preventing events and treatments given in both synthetic cohorts (4.8 net benefit in MINDACT, 3.0 in TAILORx per 1000 patients). Incorporating OncotypeDX into risk assessment improved treatment allocation more than MammaPrint (+2.6 vs + 1.6 in MINDACT, +1.3 vs + 1.0 in TAILORx contexts), with substantial uncertainty. Using the dichotomized OncotypeDX score limited its clinical usefulness compared to using its underlying continuous score.

**Discussion:**

Both MammaPrint and OncotypeDX tests improve identifying candidates for chemotherapy among women with early breast cancer, with broadly equivalent clinical usefulness. The tests should be implemented into existing risk algorithms to maximize their clinical usefulness.

## Highlights

• Direct comparisons between MammaPrint and OncotypeDX are lacking.
• A decision analytic model showed that both tests similarly improve patient outcomes.
• Continuous versions of either test may be implemented into clinical care.

## 1 Introduction

Clinical risk algorithms, such as Adjuvant! and PREDICT, use patient and tumor characteristics to help identify those most likely to benefit from treatment in early-stage breast cancer [[1], [2], [3], [4]]. When it is unclear whether treatment benefit outweighs the side-effects of chemotherapy, genomic testing can provide additional prognostic value beyond traditional clinical factors, enabling more personalized treatment decisions [5,6].

The MammaPrint and OncotypeDX are examples of genomic tests that have accumulated substantial evidence supporting their clinical validity [7,8]. The landmark MINDACT and TAILORx trials demonstrated that the MammaPrint and OncotypeDX, respectively, help identify patients with favorable prognoses beyond traditional clinical risk factors, for whom chemotherapy does not sufficiently improve survival outcomes[[9], [10], [11]]. Based on this evidence, recent guidelines (ASCO, ESMO, St. Gallen) approved both tests to guide the choice for adjuvant chemotherapy for patients whose clinical profiles (e.g. ER+, HER2-) do not provide a clear indication to start or withhold chemotherapy[[12], [13], [14]]. Although both tests have been approved, these guidelines advise using only one test per patient, without a clear preference for either.

Studies comparing MammaPrint and OncotypeDX (one using commercial versions [15], others in silico reconstructions [16,17]) illustrated mostly similar performance [7,18], leaving the questions if one test should be preferred over the other, insufficiently answered. While ideally a randomized trial would assess their comparative impact on treatment decision making [19], such trials are cost heavy and time consuming [20,21]. Alternatively, decision analytic modelling allows for a model-based assessment of how these genomic tests might influence treatment decisions and patient outcomes [22,23]. The reported results from the MINDACT and TAILORx trials provided sufficiently detailed information to build such a model-based comparison.

We aimed to compare the clinical usefulness of MammaPrint and OncotypeDX tests in guiding adjuvant chemotherapy decisions for early-stage breast cancer patients populations similar to the MINDACT and TAILORx trials. We evaluated treatment decision making with Net Benefit – a weighted difference of preventing events and the number of treatments given. We hereto extend a previously developed decision-analytic model that evaluated the MammaPrint [24], by incorporating OncotypeDX and comparing both tests within the contexts of both the MINDACT and TAILORx cohorts.

## 2 Methods

### 2.1 Decision analysis

We evaluated both the MammaPrint and OncotypeDX tests in both trial cohorts to avoid a potential (‘home-field’) advantage for one test. Two synthetic cohorts were generated: one resembling the cohort of patients included in the MINDACT trial, and another resembling the TAILORx cohort. Each synthetic cohort included 1 million unique patients. To compare potential patient outcomes with and without treatment, we cloned each patient, creating digital twins: one with treatment and one without. This resulted in a total sample size of N = 2,000,000 for each synthetic cohort.

To directly compare both genomic tests in both trial cohorts, we simulated three test scores for every twin: a clinical risk score (continuous), a MammaPrint score (high/low), and an OncotypeDX score (0–100, and dichotomized into low: 0–25 vs high: 26–100 [11]). The distribution of these test scores closely matched those observed in the trials. A continuous MammaPrint version was not included due to absence of information on its continuous score distribution. In the trials, clinical risk was estimated using Adjuvant!Online and subsequently classified as high or low clinical risk. We used a continuous clinical risk score for a more detailed clinical risk assessment as would be available in current tools such as PREDICT version 3.0 [4]. The trial cohorts differed in inclusion criteria. We therefore adjusted the genomic test scores when applying them to the other trial's cohort, as the TAILORx trial included more clinical low-risk patients than the MINDACT trial (70 % [11] vs. 50 % [10]). Second, we assumed similar prognostic performance of MammaPrint or OncotypeDX when applied to the other synthetic cohort. This assumption is supported by evidence from the RxPonder trial, which demonstrated prognostic value of OncotypeDX in node-positive disease [25], a group that was excluded in TAILORx but included in MINDACT.

### 2.2 Simulating the survival outcome

The outcome was defined as 10-year distant metastases or breast cancer-related death, excluding death from unknown or known other cause. To simulate these events, we used a survival model with clinical risk, MammaPrint, OncotypeDX, and chemotherapy as predictors.

The prognostic strength for both markers was taken from their respective trials. Chemotherapy effectiveness was estimated in the MINDACT trial, whereas the TAILORx trial could not estimate treatment effectiveness for the high risk group (score>26), because all patients received chemotherapy by design. The treatment estimate from MINDACT closely aligned with the value used in the PREDICT algorithm and was therefore considered appropriate for use in the synthetic TAILORx cohort. While some studies suggest an interaction effect between the OncotypeDX (high risk scores) and chemotherapy effectiveness [11,26,27], conclusive evidence and precise estimates for this interaction are lacking [7,11,25,28]. Because the TAILORx trial did not provide an estimate for this interaction [11,29], we could not include an interaction term and accordingly assumed no predictive effect of OncotypeDX on treatment benefit. However, the effect of using different estimates for chemotherapy effectiveness are explored in the sensitivity analysis. More information on the trials, assumptions and data generating process can be found in the supplementary materials.

### 2.3 Treatment decision-making strategies

Chemotherapy decisions were based on estimated individual treatment benefit, defined as the absolute 10-year risk (of distant metastases or breast cancer-related death) reduction by giving chemotherapy. To allocate treatment, we set a treatment threshold, which reflects the minimum treatment benefit required for chemotherapy to be recommended. We realize that recent guidelines refrain from setting a specific threshold and emphasize the importance of discussing estimated treatment benefit with individual patients [13]. However, to evaluate treatment decision-making strategies, we need a specific threshold [30]. We used 5 % risk reduction as a default treatment threshold as suggested clinically relevant in the MINDACT trial [10], with alternatively 3 % in the appendix. When visualizing the clinical usefulness of the genomic tests, we presented a wide range of thresholds (0–20 %) to reflect varying patient preferences [31]. For the default 5 % treatment threshold, we assumed that all patients whose individual treatment benefit exceeded 5 % received chemotherapy. Given the treatment indication, we selected the appropriate twin from each pair (with or without chemotherapy) to count its outcome.

**Fig. 1:**
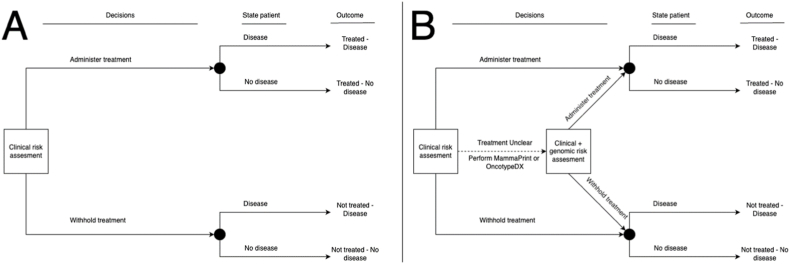
Treatment decision making with and without genomic testing. The decision to give chemotherapy is based on the 10-year absolute risk reduction from treatment, with patients treated if this reduction exceeds the treatment threshold (default: 5 %). In scenario A, treatment decisions are based solely on clinical risk assessment. Given the clinical risk, patients are treated or not, affecting their health outcomes. This allows for counting the reduction in number of events (benefit) and treatments given (harm) of this approach. In scenario B, all patients undergo clinical risk assessment first. If the risk is very high or very low, the treatment decision follows without genomic testing. If the clinical risk is intermediate, a genomic test is performed to potentially reclassify the treatment decision. Moving from A to B should lead to better treatment allocation, improving patient outcomes while avoiding overtreatment.

We focused on four treatment decision-making strategies. The clinical reference strategy was informed solely by clinical characteristics, reflecting decision-making without any genomic testing. Three strategies integrated clinical plus genomic information from either the MammaPrint, dichotomized OncotypeDX, or the continuous OncotypeDX. We explored how integrating genomic tests changed treatment decisions through better risk prediction. For some patients, treatment benefit based on their clinical risk scores was so low or high that performing a genomic test could not change the treatment decision. Therefore, genomic testing was only considered if the treatment decision based on clinical risk assessment could be reclassified by the genomic test result (Fig. 1). The four different decision-making strategies resulted in four indications to treat or not treat an individual patient.

### 2.4 Performance evaluation

To evaluate the treatment decision making strategies, we used Net Benefit as a summary measure [32,33]. As genomic tests are used to guide treatment decisions, we evaluated them on their intended use; how they affect treatment allocation and patient outcomes on the population level. Treatment benefit is defined as the number of prevented events by giving treatment, while treatment harm refers to the general burden of treatment (costs, risks of side effects) and is quantified by the number of treatments given [32]. However, to subtract the treatment harms from the benefit, a weight is needed [32].

In clinical practice, the harm of an event is considered greater than the harm of treatment. To reflect this in net benefit, a weight *w* is introduced that represents the importance of preventing one event relative to giving one treatment [32]. This weight *w* refers to the question: 'how many treatments am I willing to give to prevent one event?’, which coincides with the treatment threshold [33]. For example, if chemotherapy should reduce 10-year risk by at least 5 %, that implies accepting treating 100 patients to prevent 5 events, or treating 20 people to prevent one event. Therefore, by multiplying the number of treatments by 5 % (0.05), the weighted harms can be subtracted from the benefit: Net Benefit = Number of prevented events – number of treatment given ∗*w*, presented per 1000 patients. A higher Net Benefit indicates a more favorable balance between preventing events and the number of chemotherapy given. The clinical usefulness of a genomic test is the improvement in net benefit relative to decision making using only clinical characteristics.

### 2.5 Sensitivity analyses

To assess the robustness of our results, we varied the inputs of our decision analysis. We varied the quality of the clinical reference model and chemotherapy effectiveness over plausible ranges. To assess if using one test is significantly better than using another, we assessed the uncertainty for the difference in Net Benefit between using the MammaPrint and continuous OncotypeDX by drawing subsamples with identical sample size as the MINDACT (n = 6693) and TAILORx trial (n = 10,253). All analyses were conducted using R version 4.2.1 [34], the R-code is available in the Supplementary Material.

## 3 Results

### 3.1 Decision analysis for MINDACT context

In the synthetic MINDACT cohort, 110 out of 1000 patients were expected to experience distant metastases or breast cancer related death within 10 years if no treatment was given. In contrast, treating all 1000 patients with chemotherapy (HR = 0.64) would reduce the number of expected events to 74, preventing 36 events (Table 1). Following the MINDACT strategy, we would treat 270 patients at high clinical and high risk based on the MammaPrint (performing 501 MammaPrint tests) expecting to prevent 20 events.

**Table 1:** Expected outcomes per 1000 patients using different decision strategies for the synthetic MINDACT cohort, assuming a relative risk reduction of HR = 0.64 for chemotherapy and an absolute risk reduction threshold of 5 % to guide treatment.

	Expected events	Prevented events	Number receiving chemotherapy	Net Benefit	Genomic tests performed
***Reference strategies****:*					
*Treat none*	110	0	0	0	0
*Treat all*	74	36	1000	−13.8	0
***MINDACT strategy****:*					
*Treat C-H/G-H risk*	90	20	270	6.3	501
***Decision analytic strategies****:* *Treat if benefit >5 % based on:*					
*Clinical risk assessment, continuous*	94	16	220	4.8	0
*Clinical + binary MammaPrint*	91	19	255	6.4	389
*Clinical + binary OncotypeDX*	93	17	197	7.4	647
*Clinical + continuous OncotypeDX*	92	18	197	8.2	992

Reference strategies are treat none and treat all. The MINDACT strategy is to perform the MammaPrint genomic test for those at high clinical risk, and treat if MammaPrint shows high risk. Four decision analytic strategies treat patients based on a 5 % threshold for treatment benefit. Following every strategy, we show the expected events, prevented events, number of patients receiving chemotherapy, Net Benefit, and number of genomic tests performed per 1000 patients. Expected events shows the expected number of distant metastasis or breast cancer related deaths at 10-year (range: 74–110 per 1000). Prevented events (0–36) are due to the number of treatments given (0–1000). Genomic tests performed for the decision analytic strategies refers to those where the genomic test could reclassify the decision for chemotherapy based on only clinical risk (i.e. intermediate clinical risk). Net benefit (NB) summarizes the weighted (*w* = 0.05) balance between the prevented events and the number of treatments given, per 1000 women: NB = Prevented events – *w* ∗ Chemotherapy given (can deviate due to rounding in the table). The higher the Net Benefit, the more favorable the balance between preventing events and the number of chemotherapies given. A similar table with 3 % as treatment threshold is included in the appendix (Appendix table 2).

### 3.2 Impact of genomic testing on treatment allocation and prognosis

Assuming the 5 % treatment threshold, integrating the MammaPrint test can reclassify 39 % of the patients whose treatment benefit based on clinical characteristics lies between the 3.2 and 8.8 % (1.6–4.8 % assuming a 3 % threshold). For the binary OncotypeDX test potential reclassification can affect 65 %, namely patients in the 1.9 and 7.7 % range (1.4–5.3 % assuming a 3 % threshold). The continuous OncotypeDX test implies testing for almost all patients (99 %).

**Fig. 2:**
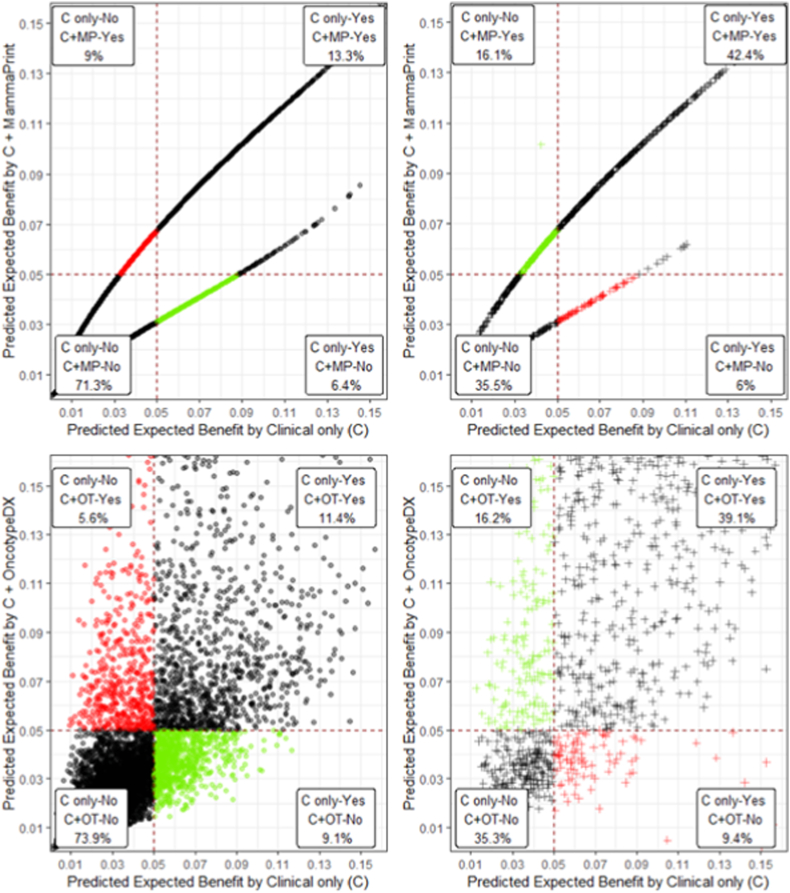
Reclassification of treatment decisions for the synthetic MINDACT cohort (5 % threshold for treatment benefit). The figures show the change in predicted treatment benefit by adding the genomic marker. The X-axis is predicted treatment benefit based on clinical risk alone, while the Y-axis shows benefit with using clinical plus genomic information. The upper two figures show reclassification by MammaPrint, and the lower figures by the continuous OncotypeDX. The figures are split by non-events (left) and events (right) at 10 years for the digital twins without treatment. A 5 % treatment threshold is used, resulting in four squares: patients where both predictions agree on the treatment indication (black), patients who are correctly reclassified by the genomic test (green) and incorrectly reclassified (red), with the percentages shown in the squares. The results show that clinical and clinical plus genomic models mostly agree, and that including a genomic marker leads to both correct and incorrect reclassifications. Abbreviations: C, clinical risk; MP, MammaPrint; OT, OncotypeDX; No/Yes, are treatment indications. (For interpretation of the references to colour in this figure legend, the reader is referred to the Web version of this article.)

Treatment benefit estimates, and thus treatment decisions, change after integrating the genomic tests (Fig. 2). Reclassification occurred in 16 % of the total patient population with the MammaPrint: 6 % were reclassified to no treatment and 10 % to receive treatment (Fig. 2). The continuous OncotypeDX also led to reclassification in 16 % of all patients, with 9 % reclassified to no treatment and 7 % to receive treatment.

### 3.3 Clinical usefulness of genomic testing

To assess the clinical usefulness of genomic testing, we counted the number of treatments given and the number of events prevented following the different treatment strategies. The Net Benefit of treating no one was 0, since there were neither treatment benefits nor harms, resulting in expecting 110 events per 1000 patients. Treating all patients resulted in a negative weighted balance between preventing events and treatments given (NB = −13.8, per 1000 patients; Table 1), as treating all patients prevented 36 events at the burden of treating 1000. Assuming at most 20 treatments per event prevented (i.e. consistent with the 5 % threshold), we would have wanted at least 50 events prevented. Following the MINDACT strategy, 20 events would be prevented with treating 270 patients, resulting in a positive Net Benefit = 20–270∗0.05 ≈ 6.3.

Treatment decision-making informed by the clinical risk alone implied treating 220 patients with a treatment threshold of 5 %, expecting to prevent 16 events, resulting in a positive Net benefit of 4.8 per 1000. Including the MammaPrint, dichotomized OncotypeDX or continuous OncotypeDX, improved the balance between treatment benefit and harms (+1.6, +2.6, +3.4 Net Benefit per 1000 patients respectively). Compared to the MammaPrint, the OncotypeDX variants tended to allocate less chemotherapy, but more genomic tests would need to be performed.

**Fig. 3:**
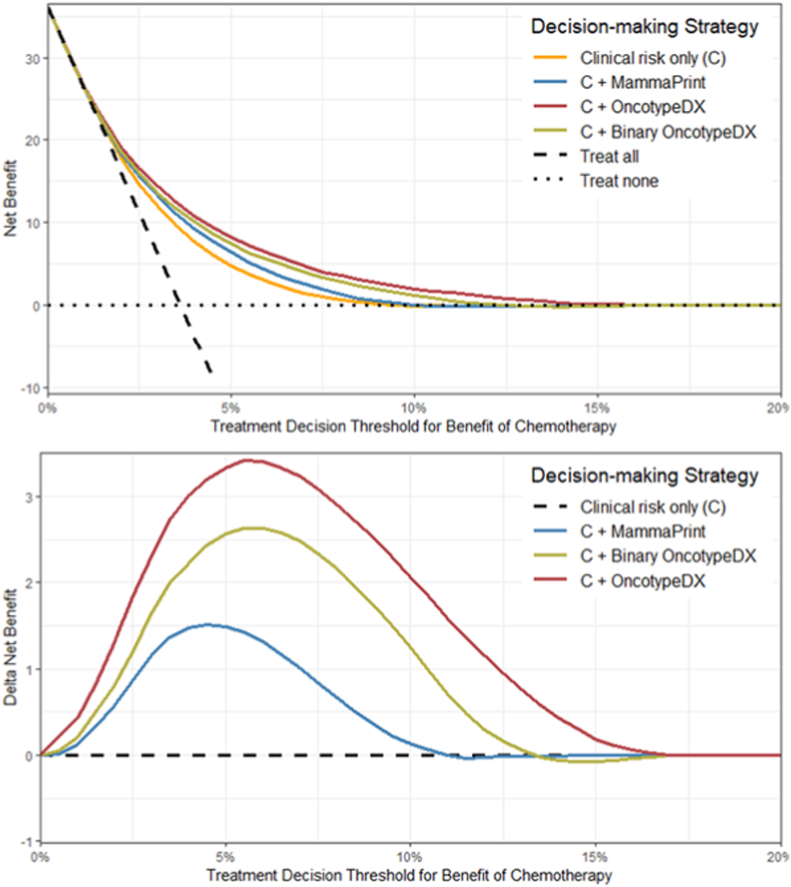
Net Benefit across different treatment decision thresholds Treatment decision-making strategies that utilize models are compared to two reference strategies (treat none, treat all) for the synthetic MINDACT cohort in the upper figure. The lower figure shows the improvement in Net Benefit (Delta Net Benefit) when adding genomic tests into clinical risk assessment, compared to using clinical risk alone. A higher Net Benefit indicates a more favorable weighted balance between prevented events and number of treatments given. The treatment threshold refers to the minimum absolute treatment benefit required to suggest treatment. A lower threshold means that one is willing to treat more patients to prevent one event. The highest Net Benefit over the range of potential treatment thresholds is observed for treatment decision-making informed by clinical risk assessment combined with the continuous OncotypeDX test, with the highest improvement relative to clinical risk assessment alone just above the 5 % threshold.

**Fig. 4:**
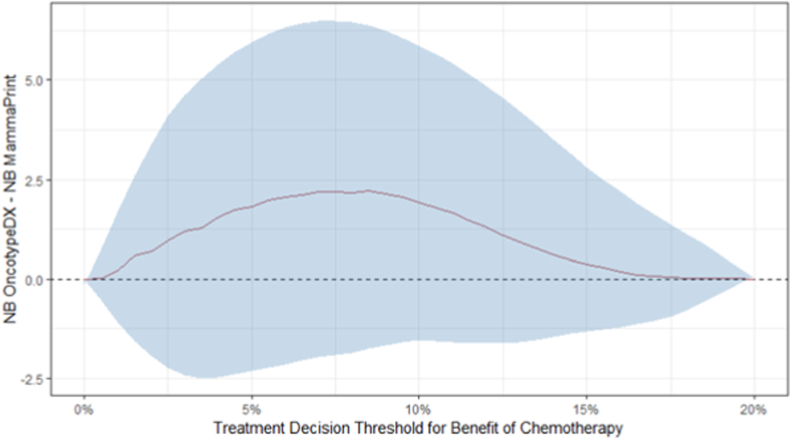
Uncertainty assessment of the difference in Net Benefit between integrating MammaPrint and OncotypeDX with clinical risk assessment across treatment thresholds, in the synthetic MINDACT cohort. The uncertainty is obtained by subsampling with an equal size to the trial size (n = 6693), with re-estimating the Net Benefit in each of 1000 the subsamples. The upper and lower bound of the 95 % confidence interval of the difference in Net Benefit are shown. The red line indicates the Net Benefit of decision-making informed by integrating the OncotypeDX into clinical risk assessment minus the Net Benefit of integrating the MammaPrint. The Net Benefit is higher for the OncotypeDX but the confidence interval includes zero, indicating no convincing outperformance. (For interpretation of the references to colour in this figure legend, the reader is referred to the Web version of this article.)

Using a 3 % treatment threshold, the improvements in Net Benefit compared to clinical risk assessment alone decreased: +1.2, +1.5, +2.4, for integrating the MammaPrint, binary OncotypeDX and continuous OncotypeDX respectively (Appendix Table 1). Across all treatment thresholds considered, the OncotypeDX tests consistently showed a slightly higher Net Benefit compared to MammaPrint, with the continuous OncotypeDX test being the highest (Fig. 3). However, the difference in Net Benefit between the continuous OncotypeDX and MammaPrint showed substantial uncertainty, precluding strong conclusions (Fig. 4).

### 3.4 Sensitivity analyses

**Appendix Fig. 1:**
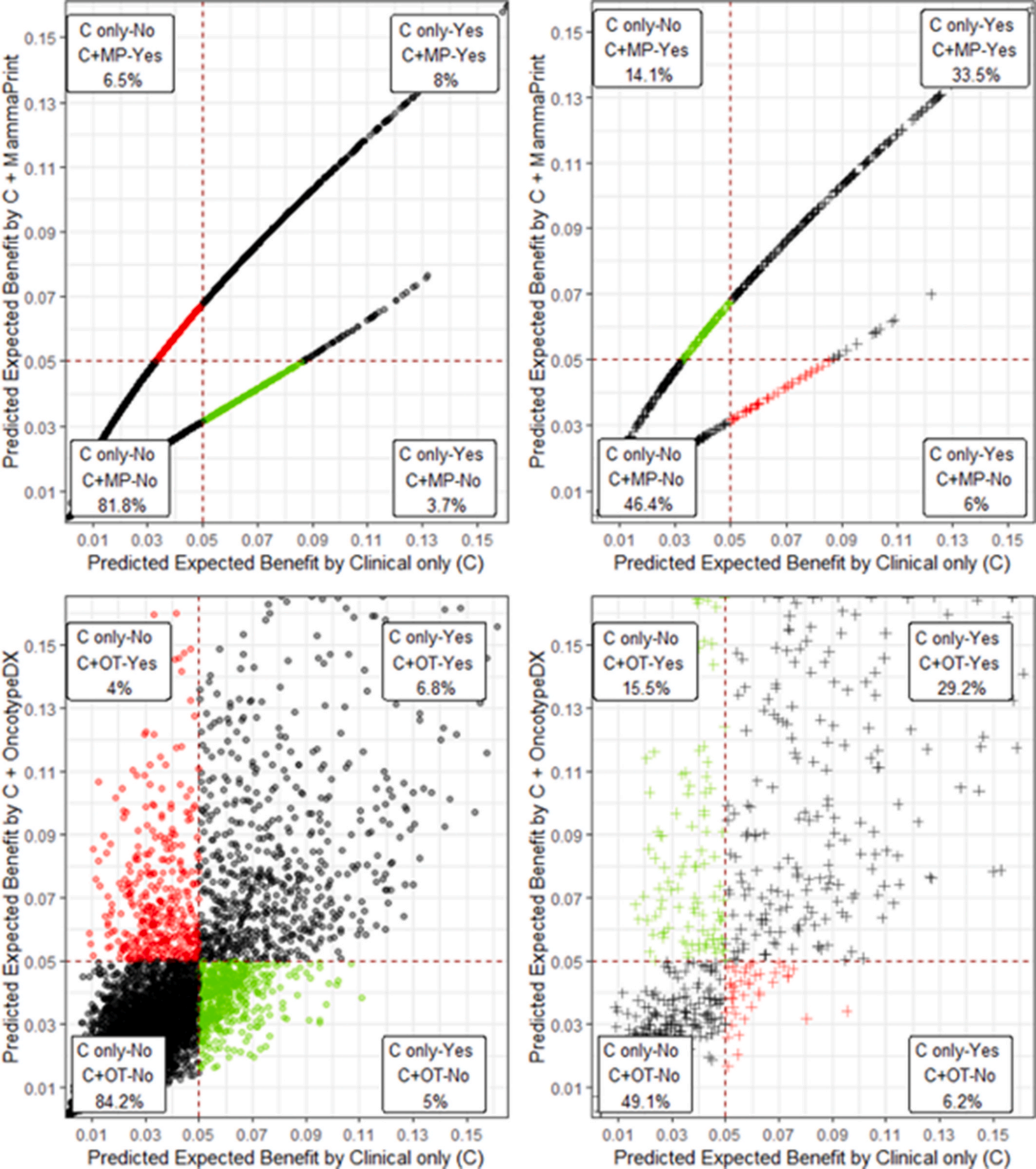
Sensitivity analysis comparing how assuming a better or worse performing reference model or chemotherapy effectiveness affects the clinical usefulness (Net Benefit) of the decision-making strategies (synthetic MINDACT cohort): The top row compares clinical risk plus the OncotypeDX versus clinical risk plus the MammaPrint. The middle row compares integrating the MammaPrint into clinical risk versus clinical risk alone, and the bottom row compares integrating the OncotypeDX in clinical risk versus clinical risk alone. On every left plot, we varied the quality of the baseline model (AUC of 0.63, 0.73, 0.82), on the right plots chemotherapy effectiveness was varied with hazard ratio (HR) = 0.64, 0.77, 0.91. Each plots shows how changes in the parameters affect the improvement of one strategy over the other.

Extensive sensitivity analyses showed that changes in inputs did not change the ranking of the MammaPrint and OncotypeDX. However, the improvements in Net Benefit by adding the genomic tests did became smaller as the clinical risk model improved, or if a less effective chemotherapy was assumed (Appendix Fig. 1).

### 3.5 Decision analysis for TAILORx context

**Appendix Fig. 2:**
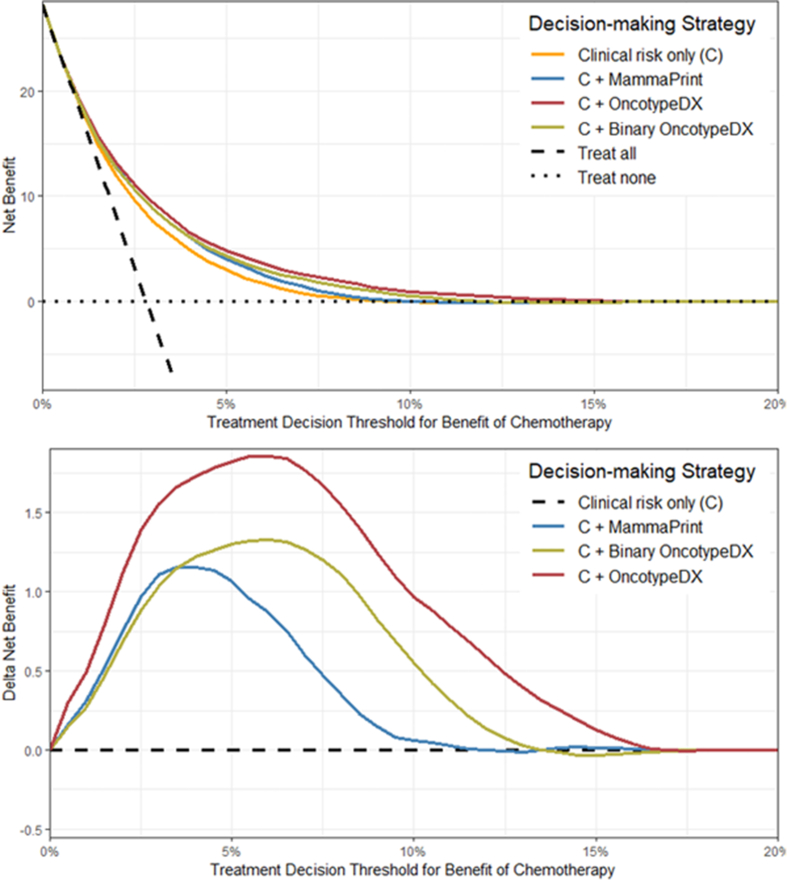
Net Benefit across different treatment decision thresholds in the synthetic TAILORx cohort Treatment decision-making strategies that utilize models are compared to two reference strategies (treat none, treat all) for the synthetic TAILORx cohort in the upper figure. The lower figure shows the improvement in Net Benefit (Delta Net Benefit) when adding genomic tests into clinical risk assessment, compared to using clinical risk alone. A higher Net Benefit indicates a more favorable weighted balance between prevented events and number of treatments given. The treatment threshold refers to the minimum absolute treatment benefit required to suggest treatment. A lower threshold means that one is more willing to treat more patients to prevent one event. The highest Net Benefit over the range of treatment thresholds is observed for treatment decision-making informed by clinical risk assessment combined with the continuous OncotypeDX test, its highest improvement relative to the clinical risk assessment alone is just above the 5 % threshold.

**Appendix Fig. 3:**
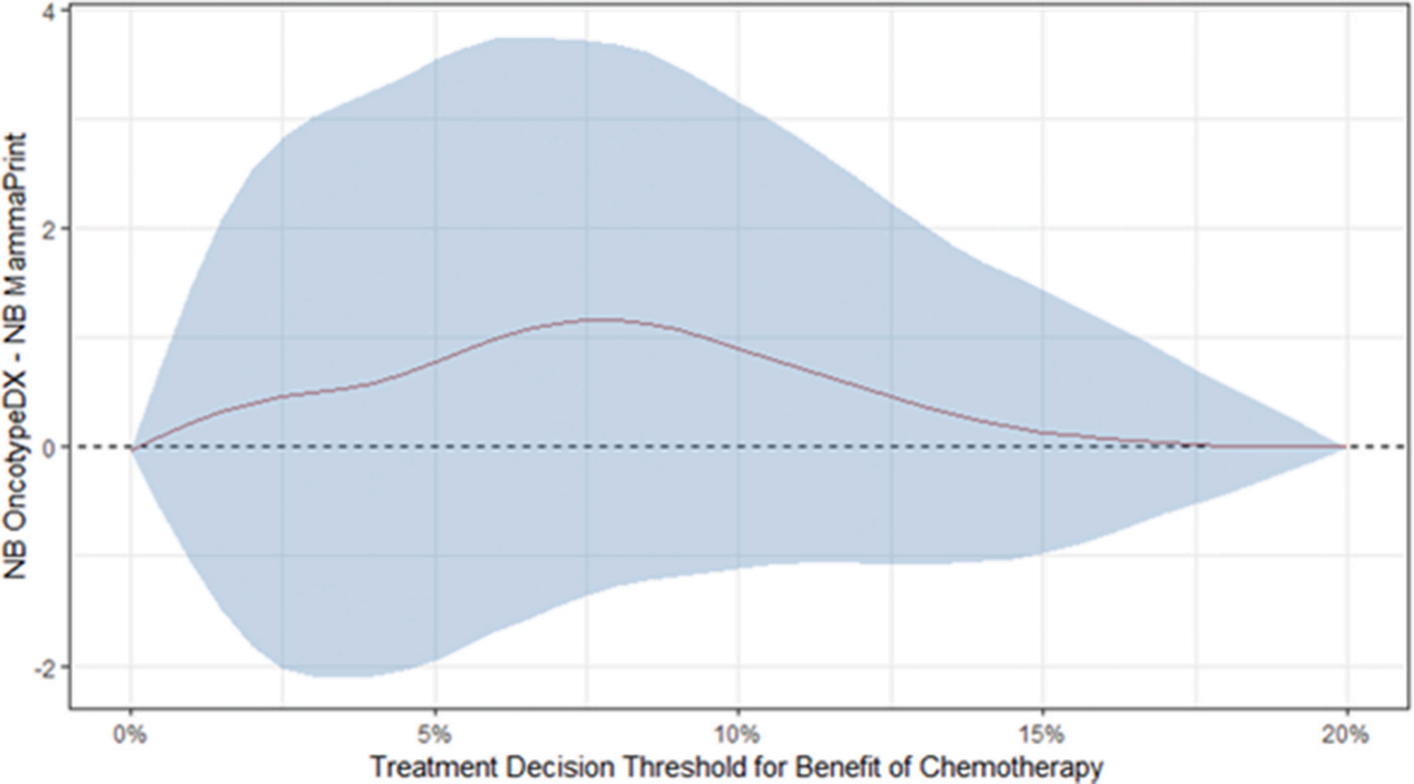
Uncertainty assessment of the difference in Net Benefit between integrating MammaPrint and OncotypeDX with clinical risk assessment across treatment thresholds, in the synthetic TAILORx cohort. The uncertainty is obtained by subsampling with an equal size to the trial size (n = 10253), with re-estimating the Net Benefit in each of 1000 the subsamples. The upper and lower bound of the 95 % confidence interval of the difference in Net Benefit are shown. The red line indicates the Net Benefit of decision-making informed by integrating the OncotypeDX into clinical risk assessment minus the Net Benefit of integrating the MammaPrint. The Net Benefit is higher for the OncotypeDX but the confidence interval includes zero, indicating no convincing outperformance.

**Appendix Fig. 4:**
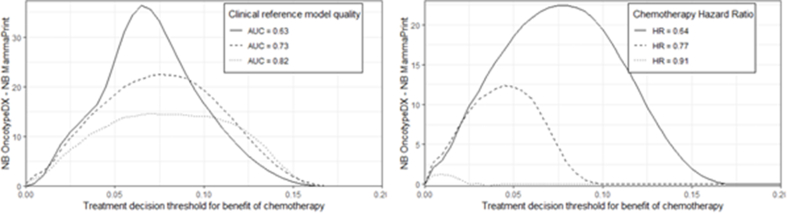
Sensitivity analysis comparing how assuming a better or worse performing reference model or chemotherapy effectiveness affects the clinical usefulness (Net Benefit) of the decision-making strategies (synthetic TAILORx cohort): The top row compares clinical risk plus the OncotypeDX versus clinical risk plus the MammaPrint. The middle row compares integrating the MammaPrint into clinical risk versus clinical risk alone, and the bottom row compares integrating the OncotypeDX in clinical risk versus clinical risk alone. On every left plot, we varied the quality of the baseline model (AUC of 0.63, 0.73, 0.82), on the right plots chemotherapy effectiveness was varied with hazard ratio (HR) = 0.64, 0.77, 0.91. Each plots shows how changes in the parameters affect the improvement of one strategy over the other.

Compared to the MINDACT context, the 10-year risk of a distant metastasis or breast cancer related death was lower in the synthetic TAILORx cohort (83 compared to 110, per 1000 patients). Treating all patients reduced the number of expected events from 83 to 55 (Appendix Table 2). Due to a lower event rate, the Net Benefit for all strategies were lower compared to the MINDACT context. Differences in Net Benefit using different genomic tests were smaller, but followed similar ranking (Appendix Tables 2 and 3). In contrast, MammaPrint had a higher Net Benefit compared to the binary OncotypeDX on certain thresholds, but only by a small margin. Again, the Net Benefit of the continuous OncotypeDX was consistently highest, but with substantial uncertainty (Appendix Figure 2 and 3). Sensitivity analysis showed similar patterns as in the MINDACT cohort (Appendix Fig. 4).

## 4 Discussion

This study provided a model based comparison of using the OncotypeDX and MammaPrint in guiding adjuvant chemotherapy decisions in populations similar to the MINDACT and TAILORx trials. We focused on how these tests affect the balance between number of chemotherapy given and preventing 10-year distant metastases or breast cancer related deaths. Both MammaPrint and OncotypeDX improved treatment allocation and patient outcomes compared to using clinical characteristics alone, supporting the claims from the MINDACT and TAILORx trials that both tests are clinically useful. Comparing their binary versions, the OncotypeDX showed a slightly more favorable balance between the number of treatments given and the number of events prevented, at the cost of needing to perform more OncotypeDX tests. Using the continuous OncotypeDX score further improved this balance, but needing to test nearly all patients (99 %) rather than testing 39 % with the MammaPrint. Since these differences in clinical impact were quite modest, our evaluations support the broadly equivalent value of these tests at the population level [7,15,18].

Despite concluding similar performance, our results provide practical information to support informed discussions about what to expect in terms of the number of treatments given, preventing events, and the number of genomic tests needed to perform. Clinical consensus should agree on what the current position of these new tests are or whether additional research is warranted. While our results illustrate potential clinical usefulness of genomic markers, their clinical usefulness can decrease when using different treatment thresholds (for example 3 % rather than 5 %), the PREDICT algorithm improves or chemotherapy is less efficient than we assumed. Notably, the way test scores were presented also influenced clinical usefulness: using the underlying continuous OncotypeDX score, rather than its standard dichotomized version, improved its clinical usefulness, though it required testing nearly all patients due to a wider reclassification range. A similar pattern is likely for MammaPrint, but could not be evaluated due to unavailable information on its continuous score.

### 4.1 Implications

To fully leverage their clinical potential, the MammaPrint and OncotypeDX tests should be integrated into established risk algorithms such as PREDICT to align with contemporary real-word clinical decision-making [16]. Since PREDICT is commonly used for clinical risk assessment, and treatment decisions are adjusted based on results of the MammaPrint or OncotypeDX test, the risk estimates from PREDICT should be updated accordingly [35]. When clinical characteristics are known and a patient specific treatment threshold is established, it becomes possible to determine whether genomic test results could result in reclassification. Women with a clear treatment benefit above or below the threshold may decide upon chemotherapy without the need of genomic testing. Further research to evaluate the performance of genomic tests in combination with PREDICT is warranted, with potential further updating of the PREDICT risk calculator [4].

Another benefit of integrating genomic tests into PREDICT is that identifying candidates for genomic testing is based on individual treatment benefit rather than group-level clinical risk profiles. Identification of clinical risk profiles that might benefit from genomic testing often relies on clinical consensus [13]. For example, ASCO guidelines recommend both the MammaPrint and OncotypeDX test for HER2 negative, ER positive, postmenopausal women with node negative or up to 3 positive nodes [12], and suggest MammaPrint primarily for those classified as clinical high risk per MINDACT. However, this dichotomization of a continuous clinical risk may overlook patients at intermediate clinical risk. We showed that patients can be reclassified in both directions by genomic test results. Once genomic tests are integrated into PREDICT, a clinician can identify whether a test might reclassify the treatment decision. For example, with a 5 % treatment threshold, MammaPrint was only informative for patients with an estimated benefit between 3.2 % and 8.8 % based on clinical characteristics. This approach reduced testing to 39 % of patients in our analysis compared to 50 % in the original MINDACT trial.

Dichotomizing continuous test scores, as is done with MammaPrint and OncotypeDX [29,36], should be approached with caution, as our findings show that how a test score is presented can influence its clinical usefulness. In our analysis, the continuous OncotypeDX had the highest effectiveness in guiding treatment decisions, but at the cost of performing more genomic tests. This raises the question about MammaPrint's potential clinical usefulness if its continuous score were utilized. Improvements of moving beyond dichotomized MammaPrint scores are already evident, such as the identification of an ultra-low-risk group [37]. Continuous scores offer more precise and individualized predictions [38], but require testing more patients. In our example, the continuous OncotypeDX would imply testing 99 % rather than 39 % of the patients. This is a considerable increase in testing costs, which would need to be evaluated in a cost-effectiveness analysis against the reduction in chemotherapy use and the number of events prevented by better targeting of chemotherapy. To mitigate extra testing, a more refined approach can be used by estimating the likelihood of a specific genomic test score based on a clinical risk profile, as not every test result is equally likely for every clinical profile.

### 4.2 Limitations

Preferably we would have access to the individual patient data to perform the analysis. With access to this data, we could have explored two potential interaction effects that might affect the effectiveness of chemotherapy. However, we did not incorporate them due to a lack of conclusive evidence.

First, some studies have suggested that high OncotypeDX scores may also predict chemotherapy benefit [26,27]. Although the TAILORx trial also hinted towards a potential interaction effect, it was not designed to formally test for an interaction between recurrence score and treatment [11]. Moreover, the RxPonder found that OncotypeDX was prognostic, but not predictive for women with ER+, HER2-, N+ and 0–25 OncotypeDX scores [25]. To date, no genomic test has demonstrated predictive capacities in a prospective design [7]. Accordingly, clinical guidelines continue to emphasize the test's prognostic role rather than its predictive ability [13,14].

Second, subgroup analyses in both MINDACT and TAILORx suggested possible interaction with menopausal status and chemotherapy effectiveness [10,11]. However, large meta-analyses (e.g., EBCTCG), have shown consistent chemotherapy effectiveness across clinical subgroups, with no indication of reduced chemotherapy benefit in women aged older than 55 years [28]. Accordingly, the PREDICT algorithm assumes that chemotherapy effectiveness is not different for pre or postmenopausal women [2,4]. Once effect estimates are available, these estimates can be used to extend further model-based comparisons by adding these potential interaction effects.

The treatment threshold is essential in identifying whom to test and whom to treat with chemotherapy. Lowering the treatment threshold from 5 % to, for example, 3 % absolute risk reduction, would result in treating more women as we are willing to treat more to prevent one event. While predicting survival outcomes is one piece of the puzzle, international projects such as 4D PICTURE (https://4DPicture.eu) emphasize the importance of patient-reported outcomes and individual preferences in establishing personalized decision-making [39]. Recent guidelines also stress the importance of discussing patient's preferences to guide treatment decisions [13]. Updates to PREDICT now include information on treatment side effects, helping patients weigh benefits and risks to establish personal treatment thresholds [40]. For our analysis, we simplified this process by using the same treatment threshold for every patient to evaluate treatment decision strategies. However, we recognize that in clinical practice individual patient preferences can lead to different treatment thresholds, with some patients willing to undergo treatment for minimal benefit and others opting for more conservative choices [31].

## 5 Conclusions

We conclude that both the MammaPrint and OncotypeDX tests improve identifying candidates for adjuvant chemotherapy among women with intermediate clinical risk. Using their continuous versions might further improve their ability to guide treatment decisions and to improve patient outcomes, however, using continuous scores comes at the cost of more genomic testing to reach their full potential. Future studies should integrate genomic tests in existing clinical decision-making models such as PREDICT to improve individualized decision-making and refinement of chemotherapy allocations.

## CRediT authorship contribution statement

**Frank Doornkamp:** Writing – review & editing, Writing – original draft, Visualization, Validation, Methodology, Investigation, Formal analysis, Conceptualization. **Liesbeth C. de Wreede:** Writing – review & editing, Writing – original draft, Visualization, Methodology, Formal analysis. **Elfi Verheul:** Writing – review & editing, Visualization. **Agnes Jager:** Writing – review & editing, Visualization. **Ewout W. Steyerberg:** Writing – review & editing, Writing – original draft, Visualization, Supervision, Methodology, Formal analysis, Conceptualization.

## Data availability statement

The code generating the data underlying this article are available in its online supplementary material.

## Funding

Funded by the European Union under Horizon Europe Work Programme 101057332. Views and opinions expressed are however those of the author(s) only and do not necessarily reflect those of the European Union or the European Health and Digital Executive Agency (HaDEA). Neither the European Union nor the granting authority can be held responsible for them. The UK team are funded under the Innovate UK Horizon Europe Guarantee Programme, UKRI Reference Number: 10041120.

**Figure:**
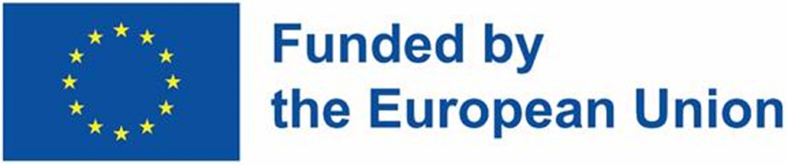

## Declaration of competing interest

The authors declare that they have no known competing financial interests or personal relationships that could have appeared to influence the work reported in this paper.

## Figures and Tables

**Appendix table 1:** Expected outcomes per 1000 patients using different decision strategies for the synthetic MINDACT cohort, assuming a relative risk reduction of HR = 0.64 for chemotherapy and a 3 % risk reduction threshold to guide treatment

	Expected events	Prevented events	Number receiving chemotherapy	Net Benefit	Genomic tests performed
***Reference strategies:***					
*Treat none*	110	0	0	0	0
*Treat all*	74	36	1000	6.2	0
***MINDACT strategy:***					
*Treat C-H/G-H risk*	90	20	270	11.7	501
***Decision analytic strategies:*** *Treat if benefit > 3 % based on*					
*Clinical risk assessment, continuous*	84	26	483	12.0	0
*Clinical + MammaPrint*	84	26	419	13.2	552
*Clinical + binary OncotypeDX*	85	25	379	13.5	636
*Clinical + OncotypeDX*	84	26	403	14.4	993

Reference strategies are treat none and treat all. The MINDACT strategy is to perform the MammaPrint genomic test for those at high clinical risk, and treat if MammaPrint shows high risk. Four decision analytic strategies treat patients based on a 3 % threshold for treatment benefit. Following every strategy, we show the expected events, prevented events, number of patients receiving chemotherapy, Net Benefit, and number of genomic tests performed per 1000 patients. Expected events shows the expected number of distant metastasis or breast cancer related deaths at 10-year (range: 74–110 per 1000). Prevented events (0–36) are due to the number of treatments given (0–1000). Genomic tests performed for the decision analytic strategies refers to those where the genomic test could reclassify the decision for chemotherapy based on only clinical risk (i.e. intermediate clinical risk). Net benefit (NB) summarizes the weighted (*w* = 0.05) balance between the prevented events and the number of treatments given, per 1000 women: NB = Prevented events – *w* ∗ Chemotherapy given (can deviate due to rounding in the table). NB is somewhat higher for the OncotypeDX test variants, which allocates less chemotherapy compared to MammaPrint, but needs more genomic testing.

**Appendix table 2:** Expected outcomes per 1000 patients using different decision strategies for the synthetic TAILORx cohort, assuming a relative risk reduction of HR = 0.64 for chemotherapy and a 5 % risk reduction threshold to guide treatment

	Expected events	Prevented events	Number receiving chemotherapy	Net benefit	Genomic tests performed
***Reference strategies:***					
*Treat none*	83	0	0	0	0
*Treat all*	55	28	1000	−21.7	0
***TAILORx strategy:***					
*Treat if OncotypeDX > 26*	73	10	136	3.4	1000
***Decision analytic strategies:*** *Treat if benefit > 5 % based on*					
*Clinical risk assessment continuous*	73	10	136	3.0	0
*Clinical + MammaPrint*	71	12	166	4.1	265
*Clinical + binary OncotypeDX*	72	11	136	4.4	431
*Clinical + OncotypeDX*	71	12	136	4.8	963

Reference strategies are treat none and treat all. The TAILORx strategy is to perform the OncotypeDX to all patients and treat those with a 26 score or higher. Four decision analytic strategies that treat based on a 5 % treatment threshold. Following every strategy, we show the expected events, prevented events, number of patients receiving chemotherapy, Net Benefit, and number of genomic tests performed per 1000 patients. Expected events shows the expected number of distant metastasis or breast cancer related deaths at 10-year (range: 55–83 per 1000). Prevented events (0–28) are due to the number of treatments given (0–1000). Genomic tests performed for the decision analytic strategies refers to those with intermediate clinical risk, where the genomic test could reclassify the decision for chemotherapy based on only clinical risk (0–100 %). Net benefit (NB) summarizes the weighted (*w* = 0.05) balance between the prevented events and the number of treatment given, per 1000 women: NB = Prevented events – *w* ∗ Chemotherapy given (can deviate due to rounding in the table). NB is negative for ‘treat all’ (−21.7), and positive for the decision analytic strategies. Integrating the genomic tests improves the NB. The OncotypeDX test variants result in somewhat higher NB more at the cost of needing to perform more tests.

**Appendix table 3:** Expected outcomes per 1000 patients using different decision strategies for the synthetic TAILORx cohort, assuming a relative risk reduction of HR = 0.64 for chemotherapy and a 3 % risk reduction threshold to guide treatment

	Expected events	Prevented events	Number receiving chemotherapy	Net benefit	Genomic tests performed
***Reference strategies:***					
*Treat none*	83	0	0	0	0
*Treat all*	55	28	100	−1.7	0
***TAILORx strategy:***					
*Treat if OncotypeDX > 26*	73	10	136	6.1	1000
***Decision analytic strategies:*** *Treat if benefit > 3 % based on*					
*Clinical only (C)*	65	18	336	7.7	0
*C + MammaPrint*	65	18	306	8.9	477
*C + binary OncotypeDX*	66	17	284	8.8	564
*C + OncotypeDX*	65	18	297	9.4	989

Reference strategies are treat none and treat all. The TAILORx strategy is to perform the OncotypeDX to all patients and treat those with a 26 score or higher. Four decision analytic strategies that treat based on a 3 % treatment threshold. Following every strategy, we show the expected events, prevented events, number of patients receiving chemotherapy, Net Benefit, and number of genomic tests performed per 1000 patients. Expected events shows the expected number of distant metastasis or breast cancer related deaths at 10-year (range: 55–83 per 1000). Prevented events (0–28) are due to the number of treatments given (0–1000). Genomic tests performed for the decision analytic strategies refers to those with intermediate clinical risk, where the genomic test could reclassify the decision for chemotherapy based on only clinical risk (0–100 %). Net benefit (NB) summarizes the weighted (*w* = 0.05) balance between the prevented events and the number of treatment given, per 1000 women: NB = Prevented events – *w* ∗ Chemotherapy given (can deviate due to rounding in the table). NB is negative for ‘treat all’ (−1.7), and positive for the decision analytic strategies. Integrating the genomic tests improves the NB. The NB for the MammaPrint and binary OncotypeDX are equivalent, with the continuous version having a slightly higher NB, but at the cost of needing to perform more tests.

**Appendix Fig. 5:**
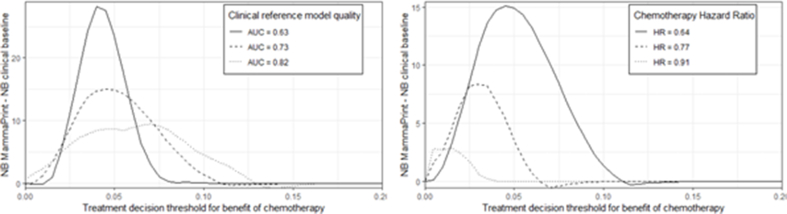


**Appendix Fig. 6:**
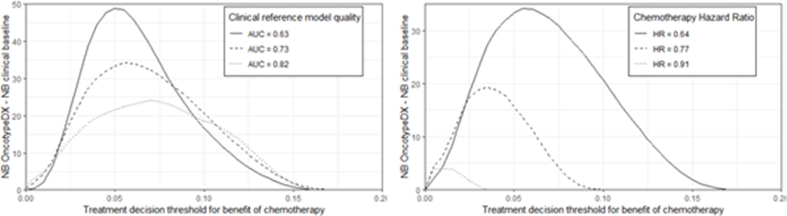


**Appendix Fig. 7:**
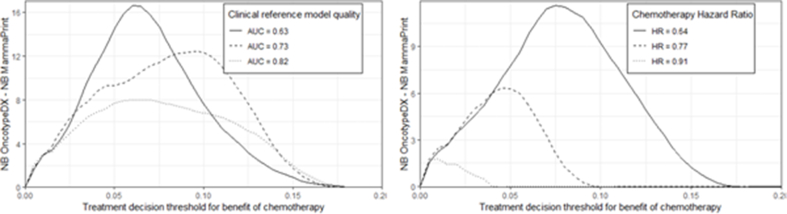


**Appendix Fig. 8:**
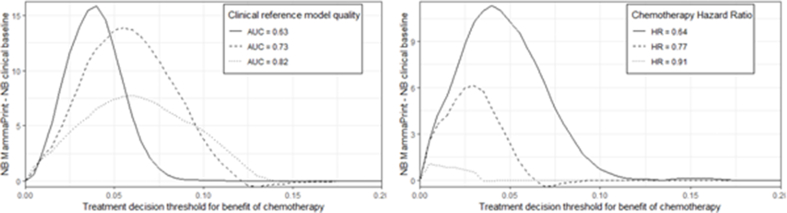


**Appendix Fig. 9:**
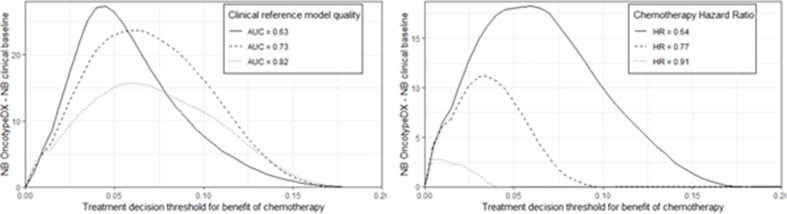


## Appendix A Supplementary data

The supplementary material includes the Rcode used to run all the analysis, and a file consisting on statistical details of the data generating mechanisms.

## References

[1] Ravdin P.M., Siminoff L.A., Davis G.J., Mercer M.B., Hewlett J., Gerson N. (2001). Computer program to assist in making decisions about adjuvant therapy for women with early breast cancer. J Clin Oncol.

[2] Candido dos Reis FJ, Wishart G.C., Dicks E.M., Greenberg D., Rashbass J., Schmidt M.K. (2017). An updated PREDICT breast cancer prognostication and treatment benefit prediction model with independent validation. Breast Cancer Res.

[3] Wishart G.C., Azzato E.M., Greenberg D.C., Rashbass J., Kearins O., Lawrence G. (2010). Predict : a new UK prognostic model that predicts survival following surgery for invasive breast cancer. Breast Cancer Res.

[4] Grootes I., Keeman R., Blows F.M., Milne R.L., Giles G.G., Swerdlow A.J. (2022). Incorporating progesterone receptor expression into the PREDICT breast prognostic model. European Journal of Cancer.

[5] Crew K.D., Hershman D.L. (2021). Better together: clinical and genomic data to inform shared decision making. J Clin Orthod.

[6] Győrffy B., Hatzis C., Sanft T., Hofstatter E., Aktas B., Pusztai L. (2015). Multigene prognostic tests in breast cancer: past, present, future. Breast Cancer Res.

[7] Blok E.J., Bastiaannet E., Van Den Hout W.B., Liefers G.J., Smit V.T.H.B.M., Kroep J.R. (2018). Systematic review of the clinical and economic value of gene expression profiles for invasive early breast cancer available in Europe. Cancer Treat Rev.

[8] Venetis K., Pescia C., Cursano G., Frascarelli C., Mane E., Camilli E.D. (2024). The evolving role of genomic testing in early breast cancer: implications for diagnosis, prognosis, and therapy. Int J Mol Sci.

[9] Cardoso F., Van’T Veer L.J., Bogaerts J., Slaets L., Viale G., Delaloge S. (2016). 70-Gene signature as an aid to treatment decisions in early-stage breast cancer. N Engl J Med.

[10] Piccart M., Van ’T Veer L.J., Poncet C., Lopes Cardozo J.M.N., Delaloge S., Pierga J.-Y. (2021). 70-gene signature as an aid for treatment decisions in early breast cancer: updated results of the phase 3 randomised MINDACT trial with an exploratory analysis by age. Lancet Oncol.

[11] Sparano J.A., Gray R.J., Makower D.F., Pritchard K.I., Albain K.S., Hayes D.F. (2018). Adjuvant chemotherapy guided by a 21-Gene expression assay in breast cancer. N Engl J Med.

[12] Andre F., Ismaila N., Allison K.H., Barlow W.E., Collyar D.E., Damodaran S. (2022). Biomarkers for adjuvant endocrine and chemotherapy in early-stage Breast Cancer: ASCO Guideline update. J Clin Oncol.

[13] Curigliano G., Burstein H.J., Gnant M., Loibl S., Cameron D., Regan M.M. (2023). Understanding breast cancer complexity to improve patient outcomes: the St Gallen International Consensus Conference for the Primary Therapy of Individuals with early Breast Cancer 2023. Ann Oncol.

[14] Loibl S., André F., Bachelot T., Barrios C.H., Bergh J., Burstein H.J. (2024). Early breast cancer: ESMO Clinical Practice Guideline for diagnosis, treatment and follow-up. Ann Oncol.

[15] Bartlett J.M.S., Bayani J., Marshall A., Dunn J.A., Campbell A., Cunningham C. (2016). Comparing breast cancer multiparameter tests in the OPTIMA prelim trial: no Test is more equal than the others. JNCI J Natl Cancer Inst.

[16] Chowdhury A., Pharoah P.D., Rueda O.M. (2023). Evaluation and comparison of different breast cancer prognosis scores based on gene expression data. Breast Cancer Res.

[17] Varga Z., Sinn P., Seidman A.D. (2019). Summary of head-to-head comparisons of patient risk classifications by the 21-gene Recurrence Score® (RS) assay and other genomic assays for early breast cancer. Int J Cancer.

[18] Harnan S., Tappenden P., Cooper K., Stevens J., Bessey A., Rafia R. (2019). Tumour profiling tests to guide adjuvant chemotherapy decisions in early breast cancer: a systematic review and economic analysis. Health Technol Assess.

[19] Steyerberg E.W., Moons K.G.M., Van Der Windt D.A., Hayden J.A., Perel P., Schroter S. (2013). Prognosis research strategy (PROGRESS) 3: prognostic model research. PLoS Med.

[20] Ferrante di Ruffano L., Davenport C., Eisinga A., Hyde C., Deeks J.J. (2012). A capture-recapture analysis demonstrated that randomized controlled trials evaluating the impact of diagnostic tests on patient outcomes are rare. J Clin Epidemiol.

[21] van Giessen A., Peters J., Wilcher B., Hyde C., Moons C., de Wit A. (2017). Systematic review of health economic impact evaluations of risk prediction models: stop developing, start evaluating. Value Health.

[22] Bossuyt P.M., Reitsma J.B., Linnet K., Moons K.G. (2012). Beyond diagnostic accuracy: the clinical utility of diagnostic tests. Clin Chem.

[23] Jenniskens K., Lagerweij G.R., Naaktgeboren C.A., Hooft L., Moons K.G.M., Poldervaart J.M. (2019). Decision analytic modeling was useful to assess the impact of a prediction model on health outcomes before a randomized trial. J Clin Epidemiol.

[24] Steyerberg E.W., De Wreede L.C., Van Klaveren D., Bossuyt P.M.M. (2021). Personalized decision making on genomic testing in early breast cancer: expanding the MINDACT trial with decision-analytic modeling. Med Decis Mak.

[25] Kalinsky K., Barlow W.E., Gralow J.R., Meric-Bernstam F., Albain K.S., Hayes D.F. (2021). 21-Gene assay to inform chemotherapy benefit in node-positive breast cancer. N Engl J Med.

[26] Albain K.S., Barlow W.E., Shak S., Hortobagyi G.N., Livingston R.B., Yeh I.-T. (2010). Prognostic and predictive value of the 21-Gene recurrence Score assay in a randomized trial of chemotherapy for postmenopausal, Node-Positive, Estrogen receptor-positive breast cancer. Lancet Oncol.

[27] Paik S., Tang G., Shak S., Kim C., Baker J., Kim W. (2006). Gene expression and benefit of chemotherapy in women with node-negative, estrogen receptor-positive breast cancer. J Clin Oncol.

[28] Braybrooke J., Bradley R., Gray R., Hills R.K., Pan H., Peto R. (2023). Anthracycline-containing and taxane-containing chemotherapy for early-stage operable breast cancer: a patient-level meta-analysis of 100 000 women from 86 randomised trials. Lancet.

[29] Sparano J.A., Gray R.J., Ravdin P.M., Makower D.F., Pritchard K.I., Albain K.S. (2019). Clinical and genomic risk to guide the use of adjuvant therapy for breast cancer. N Engl J Med.

[30] Hunink M.G.M., Weinstein M.C., Wittenberg E., Drummond M.F., Pliskin J.S., Wong J.B. (2014).

[31] Hamelinck V.C., Bastiaannet E., Pieterse A.H., Jannink I., van de Velde C.J.H., Liefers G.-J. (2014). Patients' preferences for surgical and adjuvant systemic treatment in early breast cancer: a systematic review. Cancer Treat Rev.

[32] Vickers A.J., Kattan M.W., Sargent D.J. (2007). Method for evaluating prediction models that apply the results of randomized trials to individual patients. Trials.

[33] Vickers A.J., Elkin E.B. (2006). Decision curve analysis: a novel method for evaluating prediction models. Med Decis Mak.

[34] R Core Team (2022).

[35] Moons K.G.M., van Es G.-A., Michel B.C., Büller H.R., F Habbema J.D., Grobbee D.E. (1999). Redundancy of single diagnostic Test evaluation. Epidemiology.

[36] Paik S., Shak S., Tang G., Kim C., Baker J., Cronin M. (2004). A multigene assay to predict recurrence of tamoxifen-treated, node-negative breast cancer. N Engl J Med.

[37] Lopes Cardozo J.M.N., Drukker C.A., Rutgers E.J.T., Schmidt M.K., Glas A.M., Witteveen A. (2022). Outcome of patients with an ultralow-risk 70-Gene signature in the MINDACT trial. J Clin Orthod.

[38] Royston P., Altman D.G., Sauerbrei W. (2006). Dichotomizing continuous predictors in multiple regression: a bad idea. Stat Med.

[39] Rietjens J.A.C., Griffioen I., Sierra-Pérez J., Sroczynski G., Siebert U., Buyx A. (2024). Improving shared decision-making about cancer treatment through design-based data-driven decision-support tools and redesigning care paths: an overview of the 4D PICTURE project. Palliat Care Soc Pract.

[40] Predict Breast side effects n.d. https://side-effects.breast.predict.cam/(accessed November 8, 2024).

