# Supplementary material for: Guiding treatment decisions in early breast cancer: A model-based comparison of the OncotypeDX and MammaPrint tests
Source: Breast. 2026 Jan 12;85:104698. doi: 10.1016/j.breast.2026.104698 (PMC12856286; doi:10.1016/j.breast.2026.104698)
Supplement: Multimedia component 2 [file mmc2.pdf]

Supplementary Material: Guiding Treatment Decisions in Early Breast Cancer: A Model-based comparison of the OncotypeDX and MammaPrint tests

Frank Doornkamp; Liesbeth C. de Wreede; Elfi Verheul; Agnes Jager; Ewout W. Steyerberg

December 2025

Contents

1 Introduction **3**

2 Aim of the study **3**

3 The MINDACT and TAILORx trials **3**

3.1 Assumptions for the decision analysis of the two trials 4

4 Synthetic data simulation **6**

4.1 Test scores simulation 6

4.1.1 Simulating clinical risk and MammaPrint scores in MINDACT 6

4.1.2 Simulating clinical risk and OncotypeDX scores in TAILORx 8

4.1.3 Adding both tests in both trials contexts 8

4.2 Outcome simulation 9

4.2.1 Step 1a: Replicating the MINDACT trial results 10

4.2.2 Step 1b: Replicating the TAILORx trial results 10

4.2.3 Step 2: The event generating models for both synthetic cohorts 11

5 Sensitivity Analyses **14**

6 Data analysis **14**

A Simulating competing risks **16**

A.1 Comparing risks generating mechanisms 16

1 Introduction

This file provides details on the data generating and modeling process used for the article: 'Guiding Treatment Decisions in Early Breast Cancer: A Model-based comparison of the OncotypeDX and MammaPrint tests'. For full transparency, the R code will also be made available.

This supplement is meant to communicate the statistical considerations of the analysis. The whole process can be split into simulating the synthetic cohorts and data analysis. The synthetic data simulation part can be divided into the following categories:

1. Simulating test scores
2. Simulating the outcome variable
   (a) Estimating trial-specific parameters to replicate published trial outcomes
   (b) Estimating parameters for the full event generating model

We first state the aim of the study, followed by a concise overview of the MINDACT and TAILORx trials, and highlighting some assumptions to improve comparability between the trials. Subsequently, we proceed with the steps outlined above for the data generating process and data analysis.

2 Aim of the study

We aimed to compare the clinical usefulness of two multi-genomic marker tests, MammaPrint and OncotypeDX, in guiding adjuvant chemotherapy decision-making for women with early stage breast cancer. The MINDACT [1] and TAILORx [2] trials are two landmark trials that have shown clinical validity of their multi-genomic marker tests, MammaPrint and OncotypeDX respectively. However, both trials validated only one of the genomic tests, thus having only one genomic test result for each patient. To allow for a comparison between the tests within the same population, we used a decision analytic modeling approach. We combined the results from the MINDACT and TAILORx trials to construct two synthetic patient cohorts that included both genomic tests results for all patients in both trial settings. The clinical usefulness of the genomic tests was determined by assessing the improved treatment allocation compared to using only clinical risk assessment, quantified with Net Benefit. Net Benefit is a decision analytic measure that quantifies the balance between the benefits and harms of treatment decision strategies.

3 The MINDACT and TAILORx trials

Two synthetic patient cohorts were constructed based on the MINDACT and TAILORx trials. The cohorts included both genomic tests results in both trial

settings. The Microarray in Node Negative and 1 to 3 Positive Lymph Node Disease May Avoid Chemotherapy trial (MINDACT) enrolled 6,653 women to investigate whether adding the MammaPrint to standard clinical characteristics could lead to improved selection of women for chemotherapy. The MammaPrint classified patients as low (G-low) or high (G-high) risk for distant metastasis[3, 1, 4]. Clinical risk was assessed using a dichotomized version of Adjuvant!Online, defining low and high-risk groups: C-low and C-high. Women categorized as low risk on both tests (C-low, G-low) received endocrine therapy alone, while those at high risk on both (C-high, G-high) received adjuvant chemotherapy. Women with discordant results (C-Low/G-High or C-High/G-Low) were randomized to receive adjuvant chemotherapy or not. The analysis focused on women with high clinical but low genomic risk (C-High/G-Low) who did not receive chemotherapy. The C-High/G-Low patients without chemotherapy had a 5-year distant metastasis-free survival (DMFS) of 95.1% (CI: 93.1-96.6; n = 644), meeting the non-inferiority threshold of at least 92%. Receiving additional chemotherapy in this group resulted only in a 0.9% higher 5-year DMFS. The 8-year follow-up results showed a 2.6% benefit from chemotherapy, which was below the clinically relevant threshold of 5%. These results supported that the MammaPrint test could identify women with C-high risk, ER+ breast cancer that do not benefit sufficiently from chemotherapy.

The Trial Assigning Individualized Options for Treatment (TAILORx) enrolled 10,253 women to evaluate the benefit of chemotherapy in women with intermediate scores on the OncotypeDX assay [5, 2, 6]. The OncotypeDX is a 21-gene-expression test that provides prognostic information regarding the risk of distant recurrence, expressed as a score from 0 to 100. Women with intermediate scores (11-25; n = 6711) were randomly assigned to receive either endocrine therapy alone or endocrine therapy in combination with adjuvant chemotherapy. Similar to MINDACT, the same classification tree was used to classify patients as clinically high or low risk. Endocrine therapy alone was non-inferior to the combined treatment, with a 9-year distant recurrence-free interval of 94.5% for those receiving endocrine therapy alone compared to 95.0% for those receiving additional chemotherapy. This difference of only 0.5% suggested that adjuvant chemotherapy was not sufficiently beneficial for patients with intermediate OncotypeDX scores.

3.1 Assumptions for the decision analysis of the two trials

The two trials differ in outcome definition and study population. Therefore, we had to make assumptions to make the trials comparable. Table 1 gives an overview of the differences between the MINDACT and TAILORx trials.

First, the MINDACT definition of distant metastasis free interval (DMFI) outcome is slightly different from TAILORx (distant recurrence free interval; DRFI). The MINDACT definition refers to the (old) DRFI-unk STEEP definition [1], including distant recurrences, breast cancer related death, and death due to unknown cause. The TAILORx definition follows the current STEEP definition of DRFI [7], which includes only distant recurrences and breast cancer

Table 1: Differences between the MINDACT and TAILORx trials

	MINDACT	TAILORx
Outcome definition	Distant metastasis or breast cancer related death (including death of unknown cause; DMFI)	Distant recurrence or death with distant recurrence if death is the first manifestation (DRFI)
Number of events	distant metastasis: 447 (6.6%), death (BC-related or unknown): 54 (0.8%)	distant recurrence: 307 (3.0%), death (Unclear whether BC-related or un-/known): 163 (1.6%)
Proportion clinical high risk	50%	30%
Inclusion criteria	Up to 3 lymph node positive breast cancers, both HER2 positive and negative	Lymph node negative, and HER2 negative breast cancers
Published results follow up time	8 years	9 years
Treatment effect estimate	(pooled) HR = 0.64	HR = 0.91

related death, excluding death due to unknown cause. MINDACT reported 54 breast cancer-related or unknown deaths in their DMFI endpoint ($n = 501$). Since only a small proportion of unknown deaths are observed in MINDACT's DMFI endpoint, we assumed that the models fitted on the outcomes are comparable. Throughout the file we will refer to events as including distant metastasis or breast cancer related deaths. Since this definition excludes deaths due to other (and unknown) reasons, we simulated the MINDACT setting with taking death due to another reason as a competing event (Appendix A). The results showed modest effects on our outcomes and therefore we concluded that adjusting for the competing risk was not necessary.

The observed differences in proportion of clinically high risk (50% in MINDACT and 30% in TAILORx) could be explained by differences in inclusion criteria. We accounted for these differences in clinical context by trial specific baseline risk estimation.

The MINDACT trial published 8-year follow-up results, whereas TAILORx reported 9-year follow-up results. To improve comparability, we used the more commonly used survival outcome at 10-years. No data was available to estimate the distribution of the baseline hazard for both trials. Therefore, we extrapolated our simulated results to 10 year survival probabilities, by assuming that

the baseline hazard is constant (i.e. follows an exponential distribution).

Finally, the treatment effect estimates from both trials differ substantially. The treatment effect estimated from TAILORx on the (randomized) intermediate risk group was HR = 0.91, while the estimate from MINDACT on the discordant (randomized) groups was HR = 0.64 (pooled). In the TAILORx trial, the high risk group based on OncotypeDX was assumed to have benefit from chemotherapy, suggesting an interaction effect [2]. However, no formal testing was available, missing an estimate of the interaction effect to be used in the analysis. Therefore, we assumed an equal relative risk reduction by treatment for all patients, consistent with evidence from recent meta-analyses [8], with the estimated treatment effect in MINDACT being broadly in line with these meta-analytic estimates [9]. Therefore, we used the hazard ratio observed in MINDACT for both trials. In the sensitivity analysis a scenario assuming a relative treatment effect of HR = 0.91 was included.

4 Synthetic data simulation

We used the published results from the MINDACT and TAILORx trials to construct two synthetic patient cohorts. One synthetic cohort resembled the MINDACT trial, and another resembled the TAILORx trial. Both synthetic cohorts contained 1 million ($N = 1,000,000$) unique patients each, to minimize sampling variability. Every unique patient was cloned, creating digital twins: one with treatment and one without treatment. This allowed for comparing potential patient outcomes with and without chemotherapy. In both synthetic cohorts, we generated three test scores for every twin: a clinical risk score, a MammaPrint score and an OncotypeDX score. No censoring mechanism was used.

4.1 Test scores simulation

To simulate the data, we need information about the distribution of the test scores and the correlation between the tests. The data is simulated with a multivariate standard normal distribution with given correlation matrix. The distributions from the standard normal distribution were transformed to their distributions as published in the trials (more details in the published code). However, for the clinical risk score, both trials used the same binary classification tree to dichotomize a continuous clinical risk score originally provided by the Adjuvant!Online. To allow for a more personalized risk estimate, we assumed a standard normal distribution for our clinical risk variable, representing the risk scores as currently presented in the PREDICT tool (Version 3.0).

4.1.1 Simulating clinical risk and MammaPrint scores in MINDACT

The MINDACT trial only published the dichotomized scores for the MammaPrint, not its underlying continuous score distribution. Therefore, simulating the

MammaPrint score distribution, we dichotomized the normal distribution, ensuring that dichotomization replicated the observed distribution in the MINDACT trial (64/36%; Table ??). To reflect the correlation between clinical risk and MammaPrint, we replicated the higher proportion of high MammaPrint results among clinically high-risk patients (Supp. Table S4 in [1].

Table 2: Comparison of the risk group proportions published in MINDACT ($n = 6693$) and the synthetic MINDACT cohort

Risk group	Published	Simulated	Difference
C-L, G-L	0.41	0.41	0.00
C-L, G-H	0.09	0.09	0.00
C-H, G-L	0.23	0.23	0.00
C-H, G-H	0.27	0.27	0.00

Abbreviations: C; (dichotomized) Clinical risk score, G; MammaPrint score, L; Low, H; High

4.1.2 Simulating clinical risk and OncotypeDX scores in TAILORx

The TAILORx provided information about the continuous OncotypeDX score distribution. The published OncotypeDX score distribution was closely approximated by a mixture of two beta distributions [2] (Results in Table 3). A binary version of the OncotypeDX is used with high risk defined as a score on the OncotypeDX bigger than 26.

Table 3: Simulated distribution of OncotypeDX scores for TAILORX and MINDACT settings

	0-5	5-10	10-15	15-20	20-25	25-30	30-35	35-40	40-50	50-100
TAILORx:										
Published trial cohort	0.05	0.12	0.25	0.28	0.17	0.06	0.03	0.02	0.02	0.01
Simulated cohort	0.05	0.12	0.25	0.28	0.16	0.07	0.03	0.02	0.02	0.01
MINDACT:										
Adjusted trial cohort*	0.04	0.11	0.24	0.27	0.16	0.08	0.04	0.02	0.03	0.01
Simulated cohort	0.05	0.11	0.24	0.27	0.16	0.07	0.04	0.03	0.03	0.01

*: The distribution of OncotypeDX scores published in TAILORx is adjusted for the higher proportion of clinically high risk patients in MINDACT compared to TAILORx.

The correlation between clinical risk and OncotypeDX was estimated by replicating the higher OncotypeDX scores among the high clinical risk group, as observed in TAILORx (Supp. Table S1 in [2]; Results in table 4).

Table 4: Comparison of risk group proportions in TAILORx ($n = 9971$) and the synthetic TAILORx cohort

Risk group	Published	Generated	Difference
C-L, G-L	0.13	0.15	-0.02
C-L, G-Int	0.51	0.49	0.02
C-L, G-H	0.06	0.07	-0.01
C-H, G-L	0.04	0.02	0.02
C-H, G-Int	0.18	0.20	-0.02
C-H, G-H	0.08	0.08	0.00

Abbreviations: C; (dichotomized) Clinical risk score, G; (grouped) OncotypeDX score, L; Low, Int; Intermediate (OncotypeDX score of 11-25), H; High

4.1.3 Adding both tests in both trials contexts

To simulate clinical risk, MammaPrint and OncotypeDX together, the correlation between MammaPrint and OncotypeDX needs to be given. Since neither trial used both tests, the correlation between the clinical risk and the genomic marker found in one trial was carried over to the other trial where the genomic marker was unobserved. The correlation between the MammaPrint and OncotypeDX was estimated from an external source [10], by replicating their published agreement in classification (Results in Table 5). Additionally, our correlation estimate ($r = 0.56$) between the MammaPrint and OncotypeDX is

close to a published correlation ($r = 0.6$) observed between in silico versions of the MammaPrint and OncotypeDX ([11]; Supp. Table 2).

Table 5: Agreement in classification between OncotypeDX and MammaPrint as published in Bartlett et al. (2016) vs. our simulated agreement

Classification	Published	Generated	Difference
OT-L/Int*, MP-L	0.60	0.60	0.00
OT-L/Int*, MP-H	0.02	0.04	-0.02
OT-H, MP-L	0.24	0.22	0.02
OT-H, MP-H	0.15	0.14	0.01

*: The three groups for OncotypeDX are combined in two groups as done in the trial, combining the low and intermediate risk group.
Abbreviations: OT; OncotypeDX, MP; MammaPrint, L; Low, I; Intermediate, H; High

When applying each genomic test to the population of the other trial, their synthetic score distributions were corrected for the different proportions of clinically high-risk patients (50% in MINDACT and 30% in TAILORx, respectively). This resulted in slightly different score distributions for the MammaPrint and OncotypeDX in both trial contexts as more or less clinical risk patients would lead to slightly different distributions. The resulting input for generating the data set with the three synthetic test scores distributions for both synthetic cohorts is shown in table 6.

Table 6: Input for the generation of the test scores for both synthetic cohorts

**Input**	**MINDACT**	**TAILORx**
Clinical score	N(0,1)	N(0,1)
MammaPrint score	Low: 64%, High: 36%	Low: 71%, High: 29%
OncotypeDX score (RS)	(Adjusted) mixture of beta distributions*	Mixture of beta distributions*
Correlation clinical risk and MammaPrint	r = 0.44	r = 0.44
Correlation clinical risk and OncotypeDX	r = 0.38	r = 0.38
Correlation MammaPrint and OncotypeDX	r = 0.56	r = 0.56

Correlations are shown for the variables after transforming the distributions to the match the published scores. *: See the R-code for the parameters.

4.2 Outcome simulation

We generated the event variable for both synthetic cohorts as the status indicator of the survival function at 10 years. We assumed that the survival function follows an exponential distribution, where the predictors have a linear effect on

the log hazards. The generation of the event variable was a two-step approach. First, we replicated both trial results with only their respective genomic test. Second, we re-estimated parameters estimated in step 1 to integrate both genomic tests to generate the events for both synthetic cohorts.

4.2.1 Step 1a: Replicating the MINDACT trial results

The MINDACT trial published survival probabilities for their DMFI endpoint with 8 year follow up. To replicate these survival probabilities, we estimated the cumulative baseline hazard at 8 years and hazard ratios associated with the clinical score, MammaPrint score, and chemotherapy effectiveness. The hazard ratios of the MammaPrint (HR = 2.4) and the chemotherapy effect (pooled effect of HR = 0.64 over the discordant groups) were published [1]. We (iteratively) optimized the hazard ratio for the clinical score alongside the (cumulative) baseline hazard to closely match the published survival probabilities, keeping the hazard ratios for MammaPrint and chemotherapy fixed.

Our synthetic cohort closely reflected the published group survival probabilities, only slightly overestimating survival for the C-H, G-L group (Table 7). We were able to replicate the 8-year probability of having a distant recurrence or breast cancer related death of 7% as observed in MINDACT. The model with only the clinical risk variable and chemotherapy had a C-statistic of 0.73, which aligns with C-statistics observed in validations of the PREDICT algorithm [12]. Including the MammaPrint in the model, led to a +0.02 improvement in C-statistic.

Table 7: Comparison of survival probabilities for the 8-year DMFI endpoint in MINDACT to predictions generated from our survival model

Risk group	Published	Generated	Difference
C-L, G-L	96.7%[95.9-97.3]	96.9%	0.2
C-L, G-H	93.5%[91.0-95.3]	92.8%	-0.7
C-H, G-L	92.2%[90.6-93.5]	94.1%	1.9
C-H, G-H	87.9%[86.2-89.4]	86.6%	-1.3

The synthetic DMFI outcome was generated using the following function: $S(t = 8|X) = exp(-H_0(t = 8)exp(\beta X))$, with the following parameters; H_0(t=8yr) = 0.05, and hazard ratios for clinical risk of HR = 1.80, HR = 2.4 for MammaPrint, and HR = 0.64 for chemotherapy.

4.2.2 Step 1b: Replicating the TAILORx trial results

The TAILORx trial published survival probabilities for the DRFI endpoint with 9 year follow up. To replicate these survival probabilities, we estimated the cumulative baseline hazard at 9 years and hazard ratios associated with the clinical score, OncotypeDX score, and chemotherapy effectiveness. The hazard ratios of the binary clinical risk (HR = 2.42) and the OncotypeDX (HR = 1.08) were published for the intermediate risk group [6]. Additionally, the chemotherapy

treatment effectiveness for this randomized intermediate risk group was published (HR = 0.91) [2].

We estimated the parameters for our continuous clinical risk variable and the OncotypeDX in an iterative procedure. In every iteration, we selected the intermediate risk group and fitted a model with the dichomotized clinical risk and the OncotypeDX, minimizing the distance to the published hazard ratios. Within every iteration, the (cumulative) baseline hazard was optimized to closely match the published survival probabilities, leaving out the high genomic risk group. The high genomic risk group was excluded due to a potential interaction between the OncotypeDX and treatment effect [2]. Including the high genomic risk group led to overestimation of the survival of the low and intermediate risk groups for compensating the underestimation of survival of the high risk group (due to fixing a too low chemotherapy effect of HR = 0.91 for this group). Sensitivity analysis will assess different levels of chemotherapy effectiveness.

Our probability of an 9-year DRFI event was 7%, slightly overestimating the probability observed in the TAILORx trial (6%), due to underestimation of survival in the high risk group. The model with only the clinical risk variable and chemotherapy had a C-statistic of 0.72, similar to our MINDACT scenario. Including the OncotypeDX in the model, led to a +0.04 improvement in C-statistic.

Table 8: Comparison of survival probabilities for the DRFI endpoint in TAILORx to predictions from our simulated survival model*

	Published	Estimated	Difference
G-L	96.8% ±0.7	97.8%	1.0
G-M, no chemo	94.5% ±0.5	94.0%	-0.5
G-M, chemo	95.0% ±0.5	94.5%	-0.5
G-H	86.8% ±1.7	80.5%	-6.3

*: Plus–minus values are Kaplan–Meier estimates ±SE. The synthetic DMFI outcome was generated using the following function: $S(t = 9|X) = exp(-H_0(t = 9)exp(\beta X))$, with the following parameters; H_0(t=9yr) = 0.05, and hazard ratios for clinical risk of HR = 1.82, HR = 1.06 for the OncotypeDX, and HR = 0.91 for chemotherapy.

4.2.3 Step 2: The event generating models for both synthetic cohorts

We needed an event generating model that included both tests to allow for the comparison between both tests on the same outcome. However, our focus is on comparing models integrating clinical risk with one of the two genomic tests to a reference model based on only clinical risk scores. Congruent with step 1, we assumed that the survival function follows an exponential distribution, where the predictors have a linear effect on the log hazards. For the (cumulative) baseline hazard we assumed one time unit as 10 years. The event indicates distant metastasis or breast cancer related death evaluated at 10 years. The variables in the model are clinical risk (X_1), MammaPrint score (X_2), OncotypeDX score

(X_3) and chemotherapy (X_4):

$$S(t = 10|X) = \exp(-H_0(t = 10) \exp(\beta_1 X_1 + \beta_2 X_2 + \beta_3 X_3 + \beta_4 X_4))$$

In the trials only one test per patient is observed, making the other genomic test unobserved in that trial. Therefore, the (cumulative) baseline hazard $H_0(t = 10)$ and $\beta_1, \ldots, \beta_4$ had to be chosen in such a way that the sub-models in which either the MammaPrint or OncotypeDX was absent yielded the same parameters as given in step 1a or 1b. Additionally, it was required that if we substitute the observed genomic test for the unobserved genomic test, the hazard ratio for the unobserved genomic test aligns with the estimate from step 1a or 1b. This ensured that both genomic tests had the same prognostic strength in both synthetic cohorts in the models with only one of the genomic tests. In an iterative procedure, the cumulative baseline hazard and the coefficients were optimized in the two synthetic cohorts separately to fulfill these conditions.

The parameters for the event generating model have no direct interpretation (table 9), as this model was solely used to generate the 10-year events. Only sub-models of this model were evaluated (Table 9). Note that we chose to use the same chemotherapy effectiveness for both cohorts, rather than the trial specific values, for improved comparison (see section 3.3). The probability of an event (without chemotherapy) at 10-years in the synthetic MINDACT cohort was 11.0%, and 8.3% for the synthetic TAILORx cohort. The C-statistic for the model including only the clinical risk variable in the MINDACT cohort was 0.73, with +0.02 after including MammaPrint. Including the OncotypeDX instead of the MammaPrint led to an increase of +0.06. For the TAILORx cohort, the C-statistic for the model including only the clinical risk was 0.72, with +0.02 after including MammaPrint and +0.04 if OncotypeDX was included instead of the MammaPrint.

Table 9: Input parameters for the survival function that were used to generate the 10-year distant metastasis-free interval, along with the corresponding sub-models that guided treatment decisions in both synthetic cohorts representing the MINDACT and TAILORx trials.

	**MINDACT**	**TAILORx**
**Event generating models:**		
Baseline hazard (t = 10yr)	0.07	0.05
Clinical risk	1.5	1.6
MammaPrint	1.2	1.3
OncotypeDX	1.06	1.06
Chemotherapy effectiveness	0.62	0.63
**Models informing decision making:**		
**Clinical baseline model:**		
Clinical risk	2.18	2.32
Chemotherapy	0.65	0.64
**Clinical + MammaPrint model:**		
Clinical risk	1.80	1.91
MammaPrint	2.44	2.40
Chemotherapy	0.65	0.64
**Clinical + OncotypeDX model:**		
Clinical risk	1.67	1.83
OncotypeDX	1.06	1.06
Chemotherapy	0.64	0.64
**Clinical + Binary OncotypeDX model:**		
Clinical risk	1.85	1.99
Binary OncotypeDX	2.95	2.62
Chemotherapy	0.64	0.64

The (cumulative) baseline hazard (H_0) and hazard ratios exp(β) used to generate the 10-year distant metastasis-free interval: S(t=10yr) = $\exp(-H_0(t = 10yr) * \exp(X\beta))$ for both synthetic cohorts. Additionally, the hazard ratios of the Cox regression sub-models, fitted on the generated outcomes, are shown. These sub-models are used to estimate treatment benefit, which informs chemotherapy decision-making. Results are rounded for publication purposes

We generated survival probabilities for each digital twin and these were used to generate the status indicator of the survival function at 10 years. If the survival probability was below a draw from the uniform distribution, an event was indicated. Each twin had the same survival probability since they had the same risk scores, but draws from the uniform distribution were taken independently for the unique versions of the digital twins. No censoring was simulated.

5 Sensitivity Analyses

We performed sensitivity analyses to assess uncertainty introduced by our estimated parameters for the event generating model. We varied the clinical risk coefficient to represent the case where the clinical baseline model would have been better or worse compared to our base cases. In both synthetic cohort settings, we divided and multiplied the estimated coefficient for the clinical baseline model by 1.5, covering a plausible range for the baseline model quality. To find the new coefficient for the event generating model, we iteratively fitted the clinical baseline model and minimized the distance to the preferred coefficient (the scaled base case coefficient). For the synthetic MINDACT cohort, this resulted in hazard ratios for the clinical baseline model of HR = 1.5, 2,2, 3.3 (AUC of 0.63, 0.73, and 0.82, respectively), and HR = 1.4, 2.3, 3.4 (AUC of 0.63, 0.73, 0.82, respectively) for the synthetic TAILORx cohort. Chemotherapy effectiveness was varied between the published estimates from the MINDACT trial and the TAILORx, resulting in hazard ratio estimates of HR = 0.64, 0.77, 0.91. No re-estimation of other parameters was done for varying the chemotherapy effectiveness, since it is assumed independent of the test scores.

Finally, we assessed the impact of assuming no correlation between the MammaPrint and OncotypeDX, since the correlation was estimated from an external source. We re-estimated the parameters for the event generating models (as described in 4.2.3, step 2) under the assumption of zero correlation between MammaPrint and OncotypeDX.

6 Data analysis

We compared four treatment decision-making strategies that are informed by four models. The baseline clinical risk model that includes a single continuous clinical risk variable and a chemotherapy effect. The other three extended models additionally included either the MammaPrint, OncotypeDX, or a binary version of OncotypeDX. The clinical usefulness of a genomic test compared the decision-making strategy based on clinical information with additional genomic test versus the strategy using only clinical information.

Chemotherapy decisions were based on estimated individual treatment benefit, defined as the difference in 10-year distant metastasis-free interval with and without chemotherapy. If the estimated individual treatment benefit exceeded the treatment threshold, we assumed the patient received treatment. We used 5% risk reduction as a default treatment threshold as suggested clinically relevant in the MINDACT trial[1], with alternatively 3% in the appendix. When visualizing the clinical usefulness of the genomic tests, we presented a wide range of thresholds (0 - 20%) to reflect varying patient preferences [13]. Following the four different decision-making strategies resulted in four indications to treat an individual patient. Given the treatment indication, we selected the appropriate twin from each pair (with or without chemotherapy).

We used Net Benefit (NB) to evaluate how treatment decisions, informed by

the different risk prediction models, led to an improved weighted sum between treatment benefit and harms [14]. Treatment benefit is defined as the reduction in the number of events relative to treating no one, while treatment harm is the number of treatments given to achieve this benefit. Net Benefit hence considers the burden and potential side effects of treatment explicitly for a net evaluation of improving outcomes. Net benefit can improve by either reducing the events or by reducing the amount of chemotherapy due to better targeting. Net Benefit is estimated using the following formula [14]:

$$\text{NB} = \frac{\Delta\text{Event} - w * \Delta\text{Treated}}{N}$$

$$\begin{aligned} \Delta\text{Event} &: \text{Decrease in number of events relative to treating no one} \\ \Delta\text{Treated} &: \text{Number of Treatments given} \\ w &: \text{Weight (Treatment decision threshold)} \\ N &: \text{Number of unique individuals} \end{aligned}$$

To subtract the harms from the benefits of treatment, a weight w balances the negative consequences of treating relative to preventing one event (10-year distant metastasis or breast cancer related death). This weight directly translates to the treatment threshold. For example, a minimal risk reduction of 5% implies accepting the treatment of twenty patients to prevent one event. In other words, the consequences of missing an event are twenty times worse than a treatment. This 5% decision threshold was set for the analysis, however, since women can have different preferences for the decision threshold [13], we plotted the Net Benefit over the whole range of possible treatment decision thresholds.

To assess the uncertainty around the difference in Net Benefit between the sub-model including MammaPrint and the sub-model including the OncotypeDX, we performed subsampling from our hypothetical cohorts with the corresponding trial sizes (MINDACT: n = 6653, TAILORx: n = 10253). In every subsample, we refitted the models and calculated the difference in Net Benefit between both models.

A Simulating competing risks

The outcome in both trials are focusing on breast cancer related outcomes and ignore known deaths due to other reasons. However, censoring patients that die due to other causes informative censoring. To interpret probabilities of being event-free one should use competing risk models. For the MINDACT setting, we can make an approximation for the competing risk 'death due to another cause'.

MINDACT published distant metastasis free survival (DMFS) and distant metastasis free interval (DMFI). DMFS includes 'death due to another cause', whereas it is censored for DMFI. We argue that we can use the DMFS published survival estimates as an approximate for the competing risk estimate for the death due to other causes in DMFI.

We assume that all the variables are not associated with the outcome death due to other cause. Consequently, we only have to estimate the cumulative baseline hazard of the competing risk. We divide the published survival probability of DMFS in cumulative incidence (I) of the DMFI endpoint and the competing risk (i.e. death due to another cause):

$$S_{\text{DMFS}}(t = 8|X) = 1 - (I_{\text{DMFI}}(t = 8|X) + I_{\text{CE}}(t = 8))$$

In our optimization procedure this adds another step, after we generated the coefficients of the DMFI model. To estimate the $H_{\text{CE}}(t = 8)$, we take the mean over all values of X to make it marginal:

$$S_{\text{DMFS}}(t = 8|X) = \exp(-(H_{\text{DMFI}}(t = 8) \exp(B_1X_1+B_2X_2+B_4X_4)+(H_{\text{CE}}(t = 8))))$$

We extrapolate the result to the 10 year distant metastasis free interval. We compare the hazard ratios from the models as well as the event rates from the different event status generating methods described below:

1. Uniform: generating draws from an uniform distribution and generate the event status as 0 if the survival probability exceeds the random draw and 1 otherwise

2. Time: generating time to event for every patient and subsequently event status at 10 years

3. Competing risks (CPR): generating event status, accounting for competing risks

A.1 Comparing risks generating mechanisms

Table 10 shows that there are slight variations in the hazard ratios from the cox regressions fitted on the different event generating methods. Table 11 shows that event rates are similar across the methods. Comparing the difference in event rates with and without chemotherapy between the methods shows that

CPR has a 0.001 lower difference. These minor differences will have negligible effects on our performance outcomes and therefore we opt to use uniform draws to generate event status.

Table 10: Hazard ratios for the cox regression models with event status generated using the three different methods

	Uniform	Time	CPR*
Model 1			
Clinical risk	2.20	2.22	2.22
Chemotherapy	0.64	0.64	0.64
Model 2			
Clinical risk	1.80	1.82	1.82
MammaPrint	2.48	2.49	2.49
Chemotherapy	0.64	0.64	0.64
Model 3			
Clinical risk	1.67	1.70	1.70
OncotypeDX	1.06	1.07	1.07
Chemotherapy	0.63	0.62	0.62

*: Hazard ratios for the cox regression on the breast cancer-specific hazard.

Table 11: Event rates at 10 years generated using the different methods

	Uniform	Time	CPR*
Event rate without chemo	0.091	0.091	0.090
Event rate with chemo	0.060	0.060	0.059
Difference	0.031	0.031	0.030

*: Only the event rate in the Breast cancer-specific survival.

References

[1] Martine Piccart, Laura J Van 'T Veer, Coralie Poncet, Josephine M N Lopes Cardozo, Suzette Delaloge, Jean-Yves Pierga, Peter Vuylsteke, Etienne Brain, Suzan Vrijaldenhoven, Peter A Neijenhuis, Sylvian Causeret, Tineke J Smilde, Giuseppe Viale, Annuska M Glas, Mauro Delorenzi, Christos Sotiriou, Isabel T Rubio, Sherko Kümmel, Gabriele Zoppoli, Alastair M Thompson, Erika Matos, Khalil Zaman, Florentine Hilbers, Debora Fumagalli, Peter Ravdin, Susan Knox, Konstantinos Tryfonidis, Aleksandra Peric, Bart Meulemans, Jan Bogaerts, Fatima Cardoso, and Emiel J T Rutgers. 70-gene signature as an aid for treatment decisions in early breast cancer: updated results of the phase 3 randomised MINDACT trial with an exploratory analysis by age. *The Lancet Oncology*, 22(4):476–488, April 2021.

[2] Joseph A. Sparano, Robert J. Gray, Della F. Makower, Kathleen I. Pritchard, Kathy S. Albain, Daniel F. Hayes, Charles E. Geyer, Elizabeth C. Dees, Matthew P. Goetz, John A. Olson, Tracy Lively, Sunil S. Badve, Thomas J. Saphner, Lynne I. Wagner, Timothy J. Whelan, Matthew J. Ellis, Soonmyung Paik, William C. Wood, Peter M. Ravdin, Maccon M. Keane, Henry L. Gomez Moreno, Pavan S. Reddy, Timothy F. Goggins, Ingrid A. Mayer, Adam M. Brufsky, Deborah L. Toppmeyer, Virginia G. Kaklamani, Jeffrey L. Berenberg, Jeffrey Abrams, and George W. Sledge. Adjuvant Chemotherapy Guided by a 21-Gene Expression Assay in Breast Cancer. *New England Journal of Medicine*, 379(2):111–121, July 2018.

[3] Fatima Cardoso, Laura J. Van'T Veer, Jan Bogaerts, Leen Slaets, Giuseppe Viale, Suzette Delaloge, Jean-Yves Pierga, Etienne Brain, Sylvain Causeret, Mauro DeLorenzi, Annuska M. Glas, Vassilis Golfinopoulos, Theodora Goulioti, Susan Knox, Erika Matos, Bart Meulemans, Peter A. Neijenhuis, Ulrike Nitz, Rodolfo Passalacqua, Peter Ravdin, Isabel T. Rubio, Mahasti Saghatchian, Tineke J. Smilde, Christos Sotiriou, Lisette Stork, Carolyn Straehle, Geraldine Thomas, Alastair M. Thompson, Jacobus M. Van Der Hoeven, Peter Vuylsteke, René Bernards, Konstantinos Tryfonidis, Emiel Rutgers, and Martine Piccart. 70-Gene Signature as an Aid to Treatment Decisions in Early-Stage Breast Cancer. *New England Journal of Medicine*, 375(8):717–729, August 2016.

[4] Marc J. Van De Vijver, Yudong D. He, Laura J. Van 'T Veer, Hongyue Dai, Augustinus A.M. Hart, Dorien W. Voskuil, George J. Schreiber, Johannes L. Peterse, Chris Roberts, Matthew J. Marton, Mark Parrish, Douwe Atsma, Anke Witteveen, Annuska Glas, Leonie Delahaye, Tony Van Der Velde, Harry Bartelink, Sjoerd Rodenhuis, Emiel T. Rutgers, Stephen H. Friend, and René Bernards. A Gene-Expression Signature as a Predictor of Survival in Breast Cancer. *New England Journal of Medicine*, 347(25):1999–2009, December 2002.

[5] Soonmyung Paik, Steven Shak, Gong Tang, Chungyeul Kim, Joffre Baker, Maureen Cronin, Frederick L. Baehner, Michael G. Walker, Drew Watson, Taesung Park, William Hiller, Edwin R. Fisher, D. Lawrence Wickerham, John Bryant, and Norman Wolmark. A multigene assay to predict recurrence of tamoxifen-treated, node-negative breast cancer. *The New England Journal of Medicine*, 351(27):2817–2826, December 2004.

[6] Joseph A. Sparano, Robert J. Gray, Peter M. Ravdin, Della F. Makower, Kathleen I. Pritchard, Kathy S. Albain, Daniel F. Hayes, Charles E. Geyer, Elizabeth C. Dees, Matthew P. Goetz, John A. Olson, Tracy Lively, Sunil S. Badve, Thomas J. Saphner, Lynne I. Wagner, Timothy J. Whelan, Matthew J. Ellis, Soonmyung Paik, William C. Wood, Maccon M. Keane, Henry L. Gomez Moreno, Pavan S. Reddy, Timothy F. Goggins, Ingrid A. Mayer, Adam M. Brufsky, Deborah L. Toppmeyer, Virginia G. Kaklamani, Jeffrey L. Berenberg, Jeffrey Abrams, and George W. Sledge. Clinical and Genomic Risk to Guide the Use of Adjuvant Therapy for Breast Cancer. *New England Journal of Medicine*, 380(25):2395–2405, June 2019.

[7] Sara M. Tolaney, Elizabeth Garrett-Mayer, Julia White, Victoria S. Blinder, Jared C. Foster, Laleh Amiri-Kordestani, E. Shelley Hwang, Judith M. Bliss, Eileen Rakovitch, Jane Perlmutter, Patricia A. Spears, Elizabeth Frank, Nadine M. Tung, Anthony D. Elias, David Cameron, Neelima Denduluri, Ana F. Best, Angelo DiLeo, Lawrence Baizer, Lynn Pearson Butler, Elena Schwartz, Eric P. Winer, and Larissa A. Korde. Updated Standardized Definitions for Efficacy End Points (STEEP) in Adjuvant Breast Cancer Clinical Trials: STEEP Version 2.0. *Journal of Clinical Oncology*, 39(24):2720–2731, August 2021.

[8] Jeremy Braybrooke, Rosie Bradley, Richard Gray, Robert K. Hills, Hongchao Pan, Richard Peto, David Dodwell, Paul McGale, Carolyn Taylor, Tomohiko Aihara, Stewart Anderson, Joanne Blum, Fatima Cardoso, Xiaosong Chen, John P. Crown, Bent Ejlertsen, Thomas W. P. Friedl, Nadia Harbeck, Wolfgang Janni, Maj-Britt Jensen, Eleftherios Mamounas, Kazutaka Narui, Ulrike Nitz, Larry Norton, Joyce O'Shaughnessy, Martine Piccart, Nicholas Robert, Zhi-Ming Shao, Dennis Slamon, Joseph Sparano, Toru Watanabe, Greg Yothers, Ke-Da Yu, Richard Berry, Clare Boddington, Mike Clarke, Christina Davies, Lucy Davies, Fran Duane, Vaughan Evans, Jo Gay, Lucy Gettins, Jon Godwin, Sam James, Hui Lui, Zulian Lui, Elizabeth MacKinnon, Gurdeep Mannu, Theresa McHugh, Philip Morris, Simon Read, Ewan Straiton, Aman Buzdar, Vera J. Suman, Kelly K. Hunt, Robert C. F. Leonard, Janine Mansi, Catherine Delbaldo, Pascal Piedbois, Emmanuel Quinaux, Christian Fesl, Michael Gnant, Lidija Sölkner, Guenther Steger, Hans Petter Eikesdal, Per Eystein Lønning, Valerie Bee, Helena Fung, John Mackey, Miguel Martin, Michael Press, Evandro De Azambuja, Richard Gelber, Meredith Regan, Angelo Di Leo, Veerle Van Dooren, Jean Marie Nogaret, John Bartlett, Bingshu E. Chen, Karen Gelmon, Paul E. Goss, Mark N. Levine, Wendy Parulekar, Kath-

leen I. Pritchard, Lois Shepherd, Donald Berry, Constance Cirrincione, Lawrence N. Shulman, Eric Winer, Rebecca S. Gelman, Jay R. Harris, Craig Henderson, Charles L. Shapiro, Peer Christiansen, Marianne Ewertz, Henning T. Mouridsen, Elise Van Leeuwen, Sabine Linn, Annelot G. J. Van Rossum, Harm Van Tinteren, Erik Van Werkhoven, Lori Goldstein, Robert Gray, Wolfgang Eiermann, Luca Gianni, Pinuccia Valagussa, Jan Bogaerts, Herve Bonnefoi, Coralie Poncet, Riikka Huovinen, Heikki Joensuu, Jacques Bonneterre, Pierre Fargeot, Pierre Fumoleau, Pierre Kerbrat, Elisabeth Luporsi, Moïse Namer, Eva M. Carrasco, Miguel Angel Segui, Christoph Meisner, Sibylle Loibl, Valentina Nekljudova, Christoph Thomssen, Gunter Von Minckwitz, Sherko Kümmel, Massimo Lopez, Patrizia Vici, George Fountzilas, Georgia Koliou, Dimitrios Mavroudis, Emmanouil Saloustros, Etienne Brain, Suzette Delaloge, Stefan Michiels, Simone Mathoulin-Pelissier, Jose Bines, Roberta M. B. Sarmento, Gianni Bonadonna, Cristina Brambilla, Anna Rossi, Judith Bliss, Raoul Charles Coombes, Lucy Kilburn, Michel Marty, Dino Amadori, Francesco Boccardo, Oriana Nanni, Alessandra Rubagotti, Emanuela Scarpi, Norikazu Masuda, Masakazu Toi, Takayuki Ueno, Takashi Ishikawa, Koji Matsumoto, Shintaro Takao, Harald Sommer, Pericles Foroglou, George Giokas, D. Kondylis, Byron Lissaios, Mattea Reinisch, Keun Seok Lee, Byung-Ho Nam, Jung Sil Ro, Andrea De Matteis, Francesco Perrone, Gong Tang, Norman Wolmark, Yasuo Hozumi, Yasuo Nomura, Helena Earl, Louise Hiller, Anne-Laure Vallier, Lucia De Mastro, Macro Venturini, Thierry Delozier, Jerome Lemonnier, Anne-Laure Martin, Henri Roché, Marc Spielmann, Xiasong Chen, Kunwei Shen, Kathy Albain, William Barlow, George T. Budd, Julie Gralow, Dan Hayes, Peter Bartlett-Lee, Paul Ellis, Angelo Raffaele Bianco, Michelino De Laurentiis, Sabino De Placido, Hans Wildiers, Limin Hsu, Oleg Eremin, Leslie G. Walker, Johan Ahlgren, Carl Blomqvist, Lars Holmberg, Henrik Lindman, Lina Asmar, Stephen E. Jones, Oleg Gluz, Cornelia Liedtke, Rodrigo Arriagada, Elizabeth Bergsten-Nordström, Lisa Carey, Robert Coleman, Jack Cuzick, Nancy Davidson, James Dignam, Mitch Dowsett, Prudence A. Francis, Matthew P. Goetz, Pam Goodwin, Pat Halpin-Murphy, Catherine Hill, Reshma Jagsi, Hirofumi Mukai, Yasuo Ohashi, Lori Pierce, Philip Poortmans, Vinod Raina, Daniel Rea, John Robertson, Emiel Rutgers, Roberto Salgado, Tanja Spanic, Andrew Tutt, Giuseppe Viale, Xiang Wang, Tim Whelan, Nicholas Wilcken, David Cameron, Jonas Bergh, and Sandra M. Swain. Anthracycline-containing and taxane-containing chemotherapy for early-stage operable breast cancer: a patient-level meta-analysis of 100 000 women from 86 randomised trials. *The Lancet*, 401(10384):1277–1292, April 2023. Publisher: Elsevier.

[9] Early Breast Cancer Trialists' Collaborative Group (EBCTCG). Comparisons between different polychemotherapy regimens for early breast cancer: meta-analyses of long-term outcome among 100000 women in 123 randomised trials. *The Lancet*, 379(9814):432–444, February 2012.

[10] John M. S. Bartlett, Jane Bayani, Andrea Marshall, Janet A. Dunn, Amy

Campbell, Carrie Cunningham, Monika S. Sobol, Peter S. Hall, Christopher J. Poole, David A. Cameron, Helena M. Earl, Daniel W. Rea, Iain R. Macpherson, Peter Canney, Adele Francis, Christopher McCabe, Sarah E. Pinder, Luke Hughes-Davies, Andreas Makris, Robert C. Stein, and on behalf of the OPTIMA TMG. Comparing Breast Cancer Multiparameter Tests in the OPTIMA Prelim Trial: No Test Is More Equal Than the Others. *Journal of the National Cancer Institute*, 108(9):djw050, September 2016.

[11] A. Prat, J.S. Parker, C. Fan, M.C.U. Cheang, L.D. Miller, J. Bergh, S.K.L. Chia, P.S. Bernard, T.O. Nielsen, M.J. Ellis, L.A. Carey, and C.M. Perou. Concordance among gene expression-based predictors for ER-positive breast cancer treated with adjuvant tamoxifen. *Annals of Oncology*, 23(11):2866–2873, November 2012.

[12] Francisco J. Candido dos Reis, Gordon C. Wishart, Ed M. Dicks, David Greenberg, Jem Rashbass, Marjanka K. Schmidt, Alexandra J. van den Broek, Ian O. Ellis, Andrew Green, Emad Rakha, Tom Maishman, Diana M. Eccles, and Paul D. P. Pharoah. An updated PREDICT breast cancer prognostication and treatment benefit prediction model with independent validation. *Breast Cancer Research : BCR*, 19:58, May 2017.

[13] Victoria C. Hamelinck, Esther Bastiaannet, Arwen H. Pieterse, Ilse Jannink, Cornelis J. H. van de Velde, Gerrit-Jan Liefers, and Anne M. Stiggelbout. Patients' preferences for surgical and adjuvant systemic treatment in early breast cancer: a systematic review. *Cancer Treatment Reviews*, 40(8):1005–1018, September 2014.

[14] Andrew J Vickers, Michael W Kattan, and Daniel J Sargent. Method for evaluating prediction models that apply the results of randomized trials to individual patients. *Trials*, 8(1):14, December 2007.
